# Key regulators of lactation performance in Xinjiang Brown cattle and Chinese Holstein cattle unraveled by multi-omics integration

**DOI:** 10.3389/fvets.2026.1808447

**Published:** 2026-05-08

**Authors:** Kailun Ma, Weinire Tuerhong, Xue Li, Dan Wang, JiangKun Wang, Shengchao Ma, Menghua Zhang, Xixia Huang, Qiuming Chen, Lei Xu

**Affiliations:** College of Animal Science, Xinjiang Agricultural University, Urumqi, China

**Keywords:** Chinese Holstein cattle, integrated analysis, lactation performance, metabolome, transcriptome, Xinjiang Brown cattle

## Abstract

This study aimed to elucidate the molecular regulatory mechanisms underlying lactation performance in Xinjiang Brown cattle (XJBC) and Chinese Holstein cattle (CHC). We employed a multi-omics integrative analysis strategy to systematically elucidate these mechanisms by correlating blood transcriptomic and milk/plasma metabolomic data. For each of the four seasons (spring, summer, autumn, and winter), we compared XJBC and CHC using samples from seven individuals per breed. Transcriptome analysis revealed 124, 877, 169, and 81 differentially expressed genes (DEGs) between the two breeds in spring, summer, autumn, and winter, respectively, primarily enriched in biological pathways such as immune response, signal transduction, and metabolic processes. Metabolome analysis indicated that 78, 97, 80, and 50 differentially expressed metabolites (DEMs) were identified in milk, while 80, 173, 48, and 54 DEMs were found in plasma across the respective seasons. Integrated analysis demonstrated that DEGs and DEMs associated with the blood-milk metabolic axis were significantly enriched in signaling pathways, including galactose metabolism, NF-kappa B signaling pathway, and purine/pyrimidine metabolism; those based on the blood-plasma metabolic axis were significantly enriched in pathways such as ABC transporters and ECM-receptor interaction. The results demonstrate that the observed seasonal patterns reflect how breed differences vary across seasons rather than intrinsic seasonal responses of each breed. In conclusion, this study systematically delineates the regulatory modules of lactation performance in XJBC and CHC. The identified DEGs and DEMs provide critical targets and a theoretical foundation for deciphering their molecular mechanisms and advancing molecular breeding.

## 1 Introduction

Xinjiang is located in the heart of the Eurasian continent and boasts vast grasslands, providing unique natural conditions for the development of animal husbandry. The Xinjiang Brown cattle and the Chinese Holstein cattle are representative breeds of dual-purpose (milk and meat) and specialized dairy cattle in China, respectively, and hold significant value in the regional livestock industry. The Xinjiang Brown cattle, a dual-purpose breed independently developed in China, are characterized by cold tolerance, adaptability to roughage, strong stress resistance, and excellent milk quality (1). Under total confinement conditions, they achieve an average annual milk yield of 6,000 kg, with a milk fat percentage of 4.2%, a milk protein percentage of 3.5%, and a somatic cell count of 350,000 cells/mL. Under semi-confinement and semi-pastoral conditions, the average lactation duration of Xinjiang Brown cattle is 234 days, with a mean total milk yield of 2,320 kg (2). In contrast, the Chinese Holstein cattle, an elite dairy breed developed through long-term crossbreeding of Holstein cattle introduced in the late 19th century with indigenous Chinese yellow cattle, have become the primary dairy breed in Xinjiang due to their outstanding milk production performance (3). They achieve a milk yield of 9,958.3 kg over 305 days, with an average daily yield of 35.68 kg, a milk fat percentage of 4.03%, a milk protein percentage of 3.39%, and a somatic cell count of 207,500 cells/mL (4). The differences in breeding direction and production performance between these two breeds provide an ideal model for this study to elucidate the impact of genetic background on molecular adaptation mechanisms.

Milk is often referred to as “white blood” and is considered an important component of a healthy diet. Its nutritional components, such as lactose, proteins, and lipids, are similar to those of human milk, earning it the reputation of being “the food closest to perfection” (5, 6). Blood serves as a vital transport and communication system within the body, responsible for delivering hormones, nutrients, and signaling molecules to target organs, and participating in the regulation of physiological activities such as growth and development (7). Studies have shown that substances such as minerals, vitamins, and immunoglobulins in milk can enter the mammary gland from the blood via transporter proteins on mammary epithelial cells (8). Therefore, blood may participate in the regulation of milk formation by influencing the metabolism of substrates required for the synthesis of milk fat, milk protein, and lactose.

With the advancement of sequencing technologies, omics approaches such as proteomics (9), transcriptomics (10), and metabolomics (11) have emerged, providing important means for identifying key candidate genes and marker metabolites affecting milk production performance. Compared with single-omics analysis, the integrated analysis of transcriptomics and metabolomics can reveal the intrinsic relationships between gene expression and metabolite changes, enable cross-validation between omics layers, and thereby facilitate the identification of key regulatory pathways and target genes (12). This study focuses on Xinjiang Brown cattle and Chinese Holstein cattle. A total of 14 individuals (7 per breed) with successfully collected samples across all four seasons were included in the final analyses. Blood transcriptome sequencing was performed to screen differentially expressed genes between the two breeds across spring, summer, autumn, and winter. Concurrently, metabolomics technology was employed to identify differential metabolites in milk and plasma across different seasons. Through co-enrichment analysis, we revealed the core signaling pathways underlying phenotypic differences between the two breeds and clarified their molecular regulatory differences in seasonal adaptation, thereby providing candidate targets and a theoretical basis for molecular breeding to optimize input–output ratios and enhance economic efficiency.

## 2 Materials and methods

### 2.1 Sample collection

In this study, seven Xinjiang Brown cattle and seven Chinese Holstein cattle from Xinjiang Yanben Brown Cattle Breeding and Development Co., Ltd. were selected as research subjects. All selected individuals were healthy lactating cows in their first parity, aged 21–23 months, reared under the same environmental conditions (with appropriate temperature and humidity) and fed a total mixed ration (including concentrate, silage, hay, alfalfa, etc.), with free access to water. Sampling was conducted from November 2023 to July 2024, with milk and blood samples collected in April (spring), July (summer), November (autumn), and January (winter). At the time of sampling, all cows were in mid-lactation (90–180 days postpartum). Only individuals with successfully collected qualified samples across all four seasons were included in subsequent analyses. The lactation performance data (daily milk yield, milk fat percentage, milk protein percentage, and somatic cell count) of each Xinjiang Brown cattle and Chinese Holstein cattle across different seasons are presented in Supplementary Table S1, with breed-season means also calculated therein.

For subsequent RNA (Ribonucleic acid) extraction and related analyses, 10 mL of peripheral blood was collected from the tail vein of the selected cattle using vacuum blood collection tubes. The tubes were then centrifuged at 3500 rpm for 15 min. After removing the upper plasma layer, the buffy coat was aspirated into a 1.5 mL EP tube using a pipette, followed by the addition of 1 mL TRIzol reagent (Invitrogen, United States). The mixture was vortexed for 3 min to ensure complete lysis of the buffy coat. The lysed samples were then stored at −80 °C for total RNA extraction.

With the assistance of experienced stockmen, approximately 50 mL of milk was collected collectively from the four quarters of each cow’s udder. Of this, 45 mL was transferred into a 50 mL DHI (Dairy herd improvement) test tube and immediately analyzed post-collection using a FOSS CombiFoss 7 milk composition and somatic cell count analyzer (FOSS, Denmark) to determine milk composition and somatic cell count. Another 2 mL aliquot was dispensed into a 5 mL centrifuge tube and stored at −80 °C for subsequent metabolomic analysis.

To investigate the expression patterns of plasma metabolites across different seasons, 10 mL of peripheral blood was collected from the tail vein of the same experimental cattle into vacuum blood collection tubes. After centrifugation at 3500 rpm for 15 min, the upper plasma layer was aspirated using a Pasteur pipette, aliquoted into 1.5 mL EP tubes, and rapidly stored at −80 °C for metabolite detection.

### 2.2 RNA and metabolite extraction

Total RNA was extracted from the buffy coat samples using TRIzol according to the manufacturer’s instructions. The extracted RNA was assessed using a NanoDrop 2000 spectrophotometer (Thermo Fisher Scientific, United States) by evaluating the OD260/280 and OD260/230 ratios. RNA integrity was determined using an Agilent 2,100 Bioanalyzer (Agilent Technologies, United States) and assessed using the RIN (RNA integrity number).

**Figure 1:**
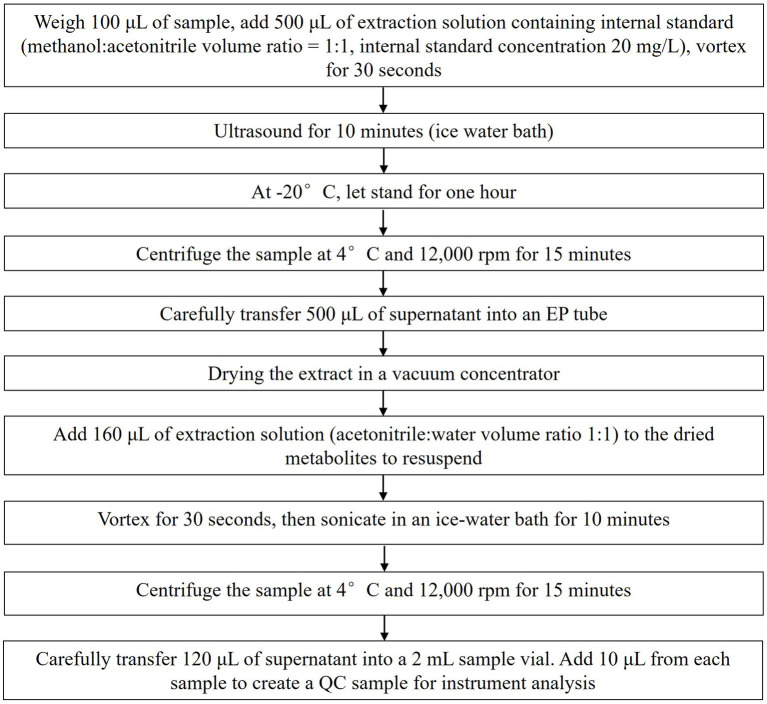
Metabolite sample extraction workflow.

Beijing Biomarker Technologies Co., Ltd., performed metabolite extraction from milk and plasma. The specific procedures are illustrated in Figure 1.

### 2.3 Sequencing library construction and RNA-Seq

Following successful quality control of the total RNA, library construction and RNA-Seq were performed by BGI Genomics Co., Ltd. Library preparation was conducted according to the sequencing platform’s manual. Briefly, mRNA was enriched from total RNA samples using oligo (dT) magnetic beads. The isolated mRNA was then fragmented to serve as templates for first-strand cDNA (Complementary deoxyribonucleic acid) synthesis with random primers. This was followed by second-strand cDNA synthesis to generate double-stranded cDNA. Subsequent steps included end repair, A-tailing, and adapter ligation. After ligation, PCR amplification was performed, and the PCR (Polymerase chain reaction) products were purified. The constructed libraries underwent quality assessment. Qualified library molecules were then circularized. The circular DNA (Deoxyribonucleic acid) molecules underwent rolling circle replication to form DNBs (DNA Nanoballs), which were finally sequenced on the DNBSEQ platform using a paired-end 150 bp (PE150) strategy.

### 2.4 Preprocessing of transcriptome data

The raw sequencing data were quality-filtered to generate high-quality, clean data for subsequent analysis. Data filtering was performed using the fastp software (13) with the following specific criteria: (1) Removal of reads with adapter sequence match ≥25% (allowing a maximum of 2 base mismatches); (2) Discarding reads shorter than 150 bp; (3) Discarding reads where the proportion of N bases was ≥0.1%; (4) Discarding reads with polyX (where X is A, T, G, or C) stretches longer than 50 bp; (5) Discarding reads where low-quality bases (quality score Q < 20) constituted ≥40% of the entire read length.

The clean data were aligned to the bovine reference genome (ARS-UCD 1.3) using the Hisat2 software (14). The effective alignment rate for all samples exceeded 94%. Based on this, the StringTie software (15) was employed, using the maximum flow algorithm and normalizing expression levels using FPKM (Fragments per kilobase of transcript per million fragments mapped) (16) as an indicator of transcript or gene expression levels. The formula for calculating FPKM is as follows:

$$\text{FPKM}=\frac{\text{cDNA Fragments}}{\text{MappedFragments}(\text{Millions})*\text{TranscriptLength}(\mathrm{kb})}$$

where cDNA Fragments denotes the number of fragments mapped to a given transcript; Mapped Fragments represents the total number of fragments mapped to all transcripts; and Transcript Length refers to the length of the transcript.

### 2.5 LC–MS/MS analysis

Metabolomic analysis was performed using an ultra-high-performance liquid chromatography-tandem high-resolution mass spectrometry (UPLC-HRMS/MS) system. The system consisted of a Waters Acquity I-Class PLUS ultra-high-performance liquid chromatograph (Waters, United States) coupled with a Waters Xevo G2-XS QTof high-resolution mass spectrometer (Waters, United States). Chromatographic separation was achieved using a Waters Acquity UPLC HSS T3 column (particle size 1.8 μm, dimensions 2.1 mm i.d. × 100 mm length). The liquid chromatography conditions were as follows: for both positive ion mode (pos) and negative ion mode (neg), mobile phase A was 0.1% formic acid in water, and mobile phase B was 0.1% formic acid in acetonitrile. The injection volume was set to 2 μL. Mass spectrometric data acquisition was conducted in MSe mode controlled by MassLynx V4.2 software (Waters, United States) to collect both primary and secondary mass spectrometry data. In this mode, mass spectral data at low (2 V) and high (10–40 V) collision energies were acquired synchronously through dual channels, with a scan rate of 0.2 s per spectrum. The ion source parameters were configured as follows: ion source temperature, 100 °C; desolvation gas temperature, 500 °C; desolvation gas flow rate, 800 L/h; cone gas flow rate, 50 L/h; cone voltage, 30 V; capillary voltage, 2,500 V for positive ion mode or −2000 V for negative ion mode; and mass-to-charge ratio (m/z) acquisition range, 50–1,200.

### 2.6 Metabolite identification and quantification

Raw metabolomic data were processed, and metabolites were identified using Progenesis QI software (17). Metabolite identification was performed by matching against the following databases: the METLIN metabolite spectral database, the KEGG (Kyoto encyclopedia of genes and genomes) pathway database, the HMDB (Human metabolome database), the Lipidmaps lipidomics database, and an in-house database from Biomarker Technologies Co., Ltd. (Beijing, China). The identification criteria were set as: a mass tolerance of 100 ppm for precursor ions and a mass tolerance of <50 ppm for fragment ions.

### 2.7 Bioinformatic and statistical analysis

#### 2.7.1 Statistical analysis of transcriptomic data

Principal component analysis (PCA) was performed on the blood transcriptome samples using the OECloud online analysis platform.^1^ DEGs between groups across seasons were identified using DESeq2 (18), with screening criteria of |Fold Change| ≥ 2 and false discovery rate (FDR) < 0.05. Heatmaps were generated for the differentially expressed genes common to all four seasons using the pheatmap package in R. Subsequently, GO (Gene Ontology, encompassing Biological Process, Cellular Component, and Molecular Function) and KEGG pathway enrichment analyses were conducted separately for the DEGs identified in each season using the DAVID online database.^2^ A term was considered significantly enriched at a threshold of *p* < 0.05.

#### 2.7.2 Statistical analysis of metabolomic data

To evaluate overall metabolic variation and screen for differential metabolites, PCA was first performed on each seasonal group using the prcomp function in R software (v3.6.1) to observe between-group separation trends and within-group sample clustering. Subsequently, orthogonal partial least squares discriminant analysis (OPLS-DA) was conducted using the ropls package to maximize inter-group discrimination. The model was constructed using 7-fold cross-validation (or several folds equal to the group sample size when the group contained fewer than 7 samples) and validated for effectiveness and reliability through 200 permutation tests. A t-test was used to calculate the significance (*p*-value) of the variation in each metabolite. R^2^Y and Q^2^Y were used to assess the predictive capability of the OPLS-DA model. Values of R^2^Y and Q^2^Y closer to 1 indicate a more stable and reliable model (suitable for screening DEMs). Generally, a model with Q^2^Y > 0.5 is considered adequate, and Q^2^Y > 0.9 indicates an excellent model. The variable importance in projection (VIP) score for each metabolite was calculated using multiple cross-validation folds. Differential expressed metabolites (DEMs) were screened using a combined criterion based on the OPLS-DA model results: |Fold Change| ≥ 1.5, VIP ≥ 1, and *p* < 0.05. Finally, functional enrichment analysis of the DEMs was performed using the KEGG database, with the *p*-value for the DEM list calculated based on the hypergeometric distribution.

#### 2.7.3 Multi-omics integrative analysis

To explore the associations between DEGs and DEMs, data from seasonal blood transcriptomics and milk/plasma metabolomics were integrated. A two-way orthogonal partial least squares (O2PLS) model combined with Pearson correlation analysis was employed for joint analysis. The Pearson correlation coefficients between all differentially expressed genes and differentially expressed metabolites in each season were calculated, and correlation heatmaps were constructed based on the top 20 differentially expressed genes and metabolites ranked by |log_2_FC| in each season; O2PLS analysis was used to reveal the overall correlation structure between the two omics datasets. Subsequently, the “Joint Pathway Analysis” function of MetaboAnalyst 6.0^3^ was used to perform KEGG co-enrichment analysis of seasonal DEGs and DEMs, thereby identifying key biological pathways at the integrative omics level. The parameters were set as follows: All pathways (integrated), Hypergeometric Test, Degree Centrality, and Combine *p*-values (unweighted). Co-enriched pathways with a *p* < 0.05 significance threshold were visualized using the online platform.^4^

## 3 Results

### 3.1 PCA analysis of blood transcriptomes in XJBC and CHC

**Figure 2:**
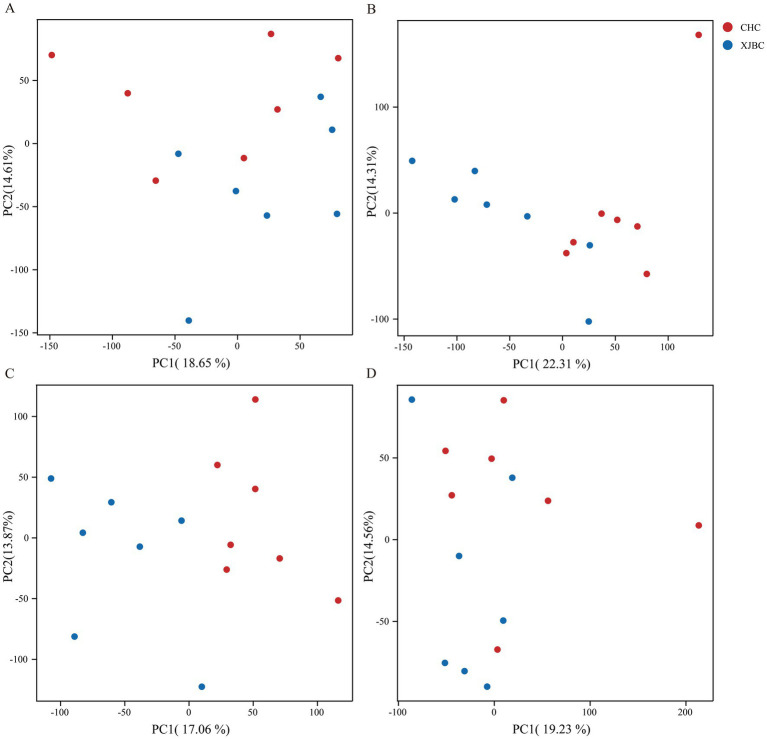
PCA analysis of blood transcriptomes in XJBC and CHC across different seasons: **(A)** Spring, **(B)** Summer, **(C)** Autumn, **(D)** Winter. In the figure, PC1 and PC2 represent the first and second principal components, respectively, and the colored dots correspond to distinct breeds.

Following sequencing quality control, an average of approximately 35.21 million clean reads and 10.6 billion clean bases were obtained per sample. Quality assessment indicated that the percentage of bases with Q20 exceeded 98% for all samples, Q30 exceeded 95%, and the GC content remained stable at around 50%, with the maximum variation between samples not exceeding 5%. These metrics confirm reliable sequencing quality and successful library construction. The average alignment efficiency of sequences from each sample to the reference genome was 91.62%, meeting the requirements for subsequent analyses. Detailed data are provided in Supplementary Tables S2, S3. PCA revealed that the blood transcriptomic data from the two cattle breeds showed a separation trend in the principal component space. In spring, samples showed some overlap but maintained a clustering trend (Figure 2A). Summer samples showed better separation, although a few samples were closely spaced (Figure 2B). Autumn samples exhibited significant separation with apparent clustering (Figure 2C). Winter samples showed a tendency to cluster, though they still overlapped to some extent (Figure 2D). Along the PC2 axis, the distinction between breeds was more pronounced in summer and autumn. Furthermore, the distribution of Xinjiang Brown cattle samples was more dispersed in spring and winter.

### 3.2 Analysis of DEGs, and GO and KEGG enrichment in XJBC and CHC

**Figure 3:**
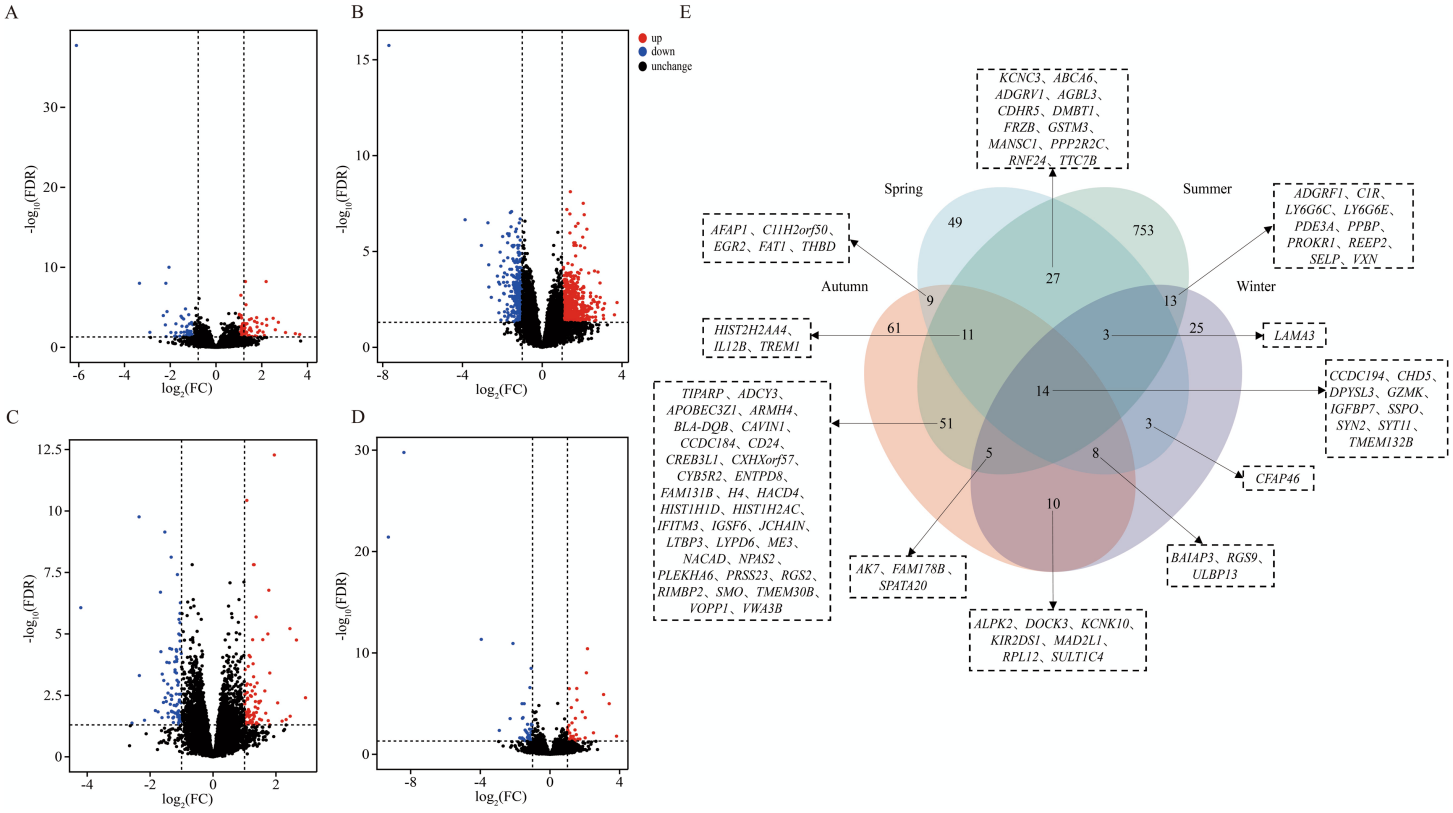
Analysis of differentially expressed genes between XJBC and CHC across different seasons. **(A)** Volcano plot of differentially expressed genes between two varieties in spring. **(B)** Volcano plot of differentially expressed genes between two varieties in summer. **(C)** Volcano plot of differentially expressed genes between two varieties in autumn. **(D)** Volcano plot of differentially expressed genes between two varieties in winter. **(E)** Venn diagram of differentially expressed genes common across the four seasons.

Differential expression analysis of RNA-Seq data revealed significant seasonal fluctuations in the blood transcriptomes of Chinese Holstein cattle and Xinjiang Brown cattle. Among the seasons, summer exhibited the most pronounced differences, with a total of 877 DEGs identified (565 upregulated and 312 downregulated; Supplementary Table S5). In contrast, the numbers of DEGs in spring, autumn, and winter were relatively lower, with 124 (76 upregulated, 48 downregulated; Supplementary Table S4), 169 (86 upregulated, 83 downregulated; Supplementary Table S6), and 81 (36 upregulated, 45 downregulated; Supplementary Table S7) DEGs, respectively (Figures 3A–D). Venn diagram analysis further revealed seasonally shared gene sets (Figure 3E). The results indicated shared DEGs across three seasons: spring–summer-autumn (11 genes), spring-autumn-winter (8 genes), spring–summer-winter (3 genes), and summer-autumn-winter (5 genes). More importantly, 14 DEGs were common across all four seasonal groups, including *CCDC194*, *CHD5*, *DPYSL3*, *GZMK*, *IGFBP7*, *SSPO*, *SYN2*, *SYT11*, and *TMEM132B*. The common differentially expressed genes exhibited clear clustering patterns of expression among individuals, with some individuals showing high expression while others showed low expression (Supplementary Figure S1).

**Figure 4:**
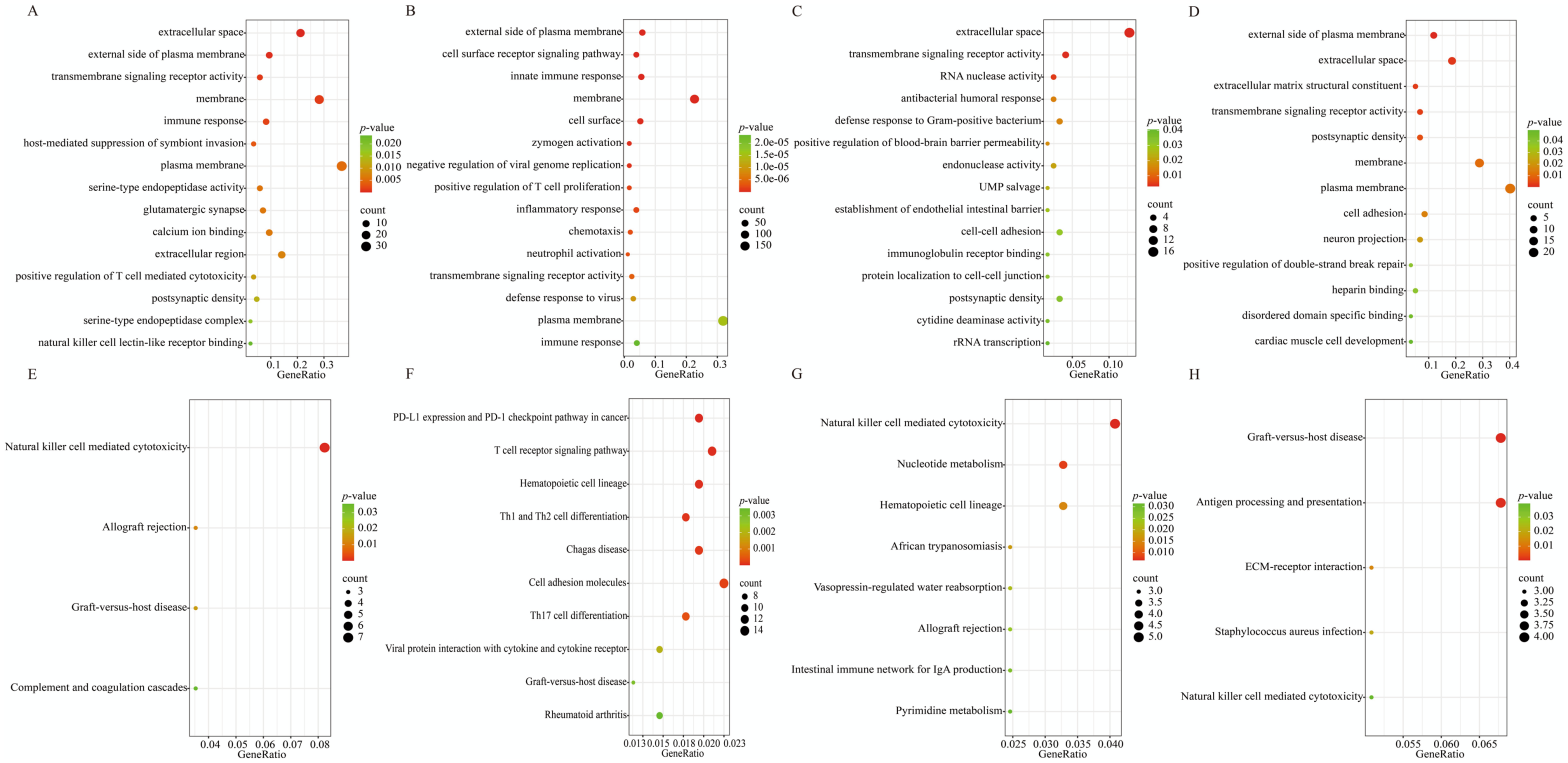
Enrichment analysis of differentially expressed genes between XJBC and CHC across different seasons. **(A)** GO enrichment analysis results of differentially expressed genes between two varieties in spring. **(B)** GO enrichment analysis results of differentially expressed genes between two varieties in summer. **(C)** GO enrichment analysis results of differentially expressed genes between two varieties in autumn. **(D)** GO enrichment analysis results of differentially expressed genes between two varieties in winter. **(E)** KEGG enrichment analysis results of differentially expressed genes between two varieties in spring. **(F)** KEGG enrichment analysis results of differentially expressed genes between two varieties in summer. **(G)** KEGG enrichment analysis results of differentially expressed genes between two varieties in autumn. **(H)** KEGG enrichment analysis results of differentially expressed genes between two varieties in winter.

GO enrichment analysis was performed on the DEGs from the spring, summer, autumn, and winter groups, respectively (Figures 4A–D). The results showed that the DEGs from the four seasons were significantly enriched in 21, 122, 15, and 13 GO terms (*p* < 0.05). In spring, the enriched terms involved Biological Processes (BP, 6 terms), Cellular Components (CC, 8 terms), and Molecular Functions (MF, 7 terms), including entries such as extracellular space, external side of plasma membrane, transmembrane signaling receptor activity, membrane, immune response, and host-mediated suppression of symbiont invasion. This suggests that the functions of differential genes in this season may primarily focus on immune defense against diseases and neuro-stress regulation. In the summer group, there were 71 BP, 21 CC, and 30 MF terms, including the external side of the plasma membrane, cell surface receptor signaling pathway, innate immune response, membrane, and cell surface. This indicates that the summer DEGs are broadly involved in processes such as immune defense and inflammation regulation, growth and development, and basic metabolism and adaptation. In the autumn group, there were 8 BP, 2 CC, and 5 MF terms, significantly enriched for entries such as extracellular space, transmembrane signaling receptor activity, RNA nuclease activity, antibacterial humoral response, and defense response to Gram-positive bacterium. This reveals that the functions of autumn DEGs may be associated with disease resistance, tissue health, and fundamental cellular functions. In the winter group, there were 3 BP, 6 CC, and 4 MF terms, including external side of plasma membrane, extracellular space, extracellular matrix structural constituent, transmembrane signaling receptor activity, and postsynaptic density. The winter DEGs may be related to growth, development, and basic signal transduction.

As shown in Figures 4E–H, the DEGs from the spring, summer, autumn, and winter groups were significantly enriched in 4, 41, 8, and 5 KEGG signaling pathways, respectively (*p* < 0.05). Among them, the spring DEGs were mainly enriched in pathways such as Natural killer cell mediated cytotoxicity, Allograft rejection, Graft-versus-host disease, and Complement and coagulation cascades, focusing on disease resistance, immune function, and tissue health and repair in cattle. The summer group had the highest number of enriched pathways, which were concentrated in basic metabolism, growth and development, and adaptive responses, including PD-L1 expression and the PD-1 checkpoint pathway in cancer, T cell receptor signaling pathway, Hematopoietic cell lineage, Th1 and Th2 cell differentiation, and Chagas disease. The autumn DEGs were significantly enriched in pathways including Natural killer cell-mediated cytotoxicity, Nucleotide metabolism, Hematopoietic cell lineage, African trypanosomiasis, and Vasopressin-regulated water reabsorption, reflecting functional characteristics related to immune defense, basic metabolic support, and regulation of physiological homeostasis. The significantly enriched pathways in the winter group included immune defense, including Graft-versus-host disease, Antigen processing and presentation, and ECM-receptor interaction.

### 3.3 Characteristics of milk metabolites and KEGG enrichment analysis of DEMs in XJBC and CHC

#### 3.3.1 Overview of milk metabolites

**Figure 5:**
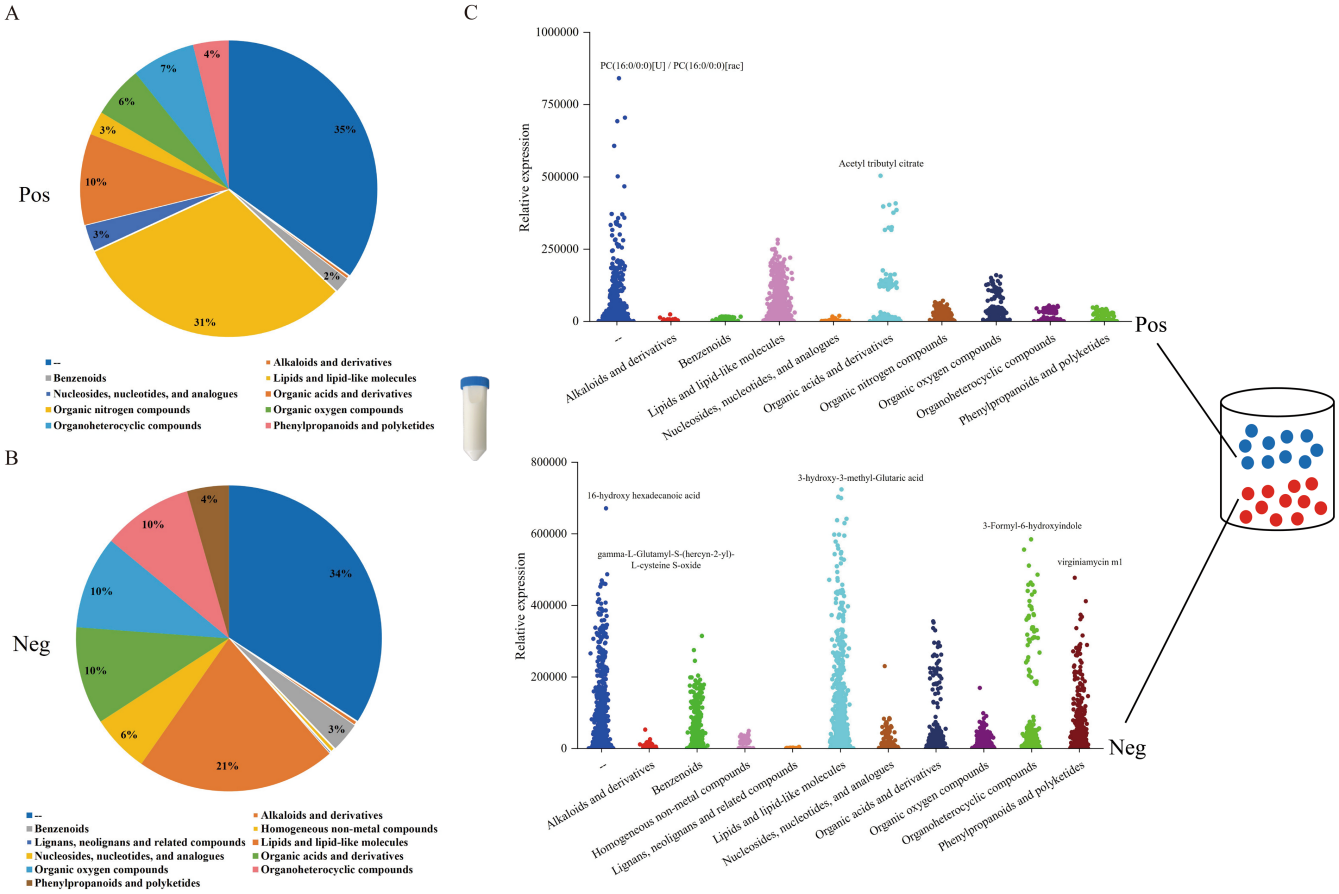
Composition of milk metabolites in XJBC and CHC across different seasons. **(A)** Composition of metabolites in positive ion mode. **(B)** Composition of metabolites in negative ion mode. **(C)** Expression characteristics of milk metabolites.

Based on the milk metabolomic data, 639 metabolites were identified in this study. Their category distribution and expression characteristics are shown in Figures 5A–C. Among them, 232 metabolites were detected in positive ion mode (pos). These were classified into 10 superclasses, 35 classes, and 56 subclasses according to the HMDB classification. Five metabolites had an average relative abundance greater than 100,000, and 35 metabolites had an average relative abundance between 10,000 and 100,000. Of these, 81 metabolites were unclassified, 72 were classified as ‘Lipids and lipid-like molecules’, 23 as ‘Organic acids and derivatives’, 16 as ‘Organoheterocyclic compounds’, 13 as ‘Organic oxygen compounds’, 9 as ‘Phenylpropanoids and polyketides’, 4 as ‘Benzenoids’, and 7 as ‘Nucleosides, nucleotides, and analogues’. In negative ion mode (neg), 407 metabolites were detected. These were classified into 11 HMDB superclasses, 61 HMDB classes, and 91 HMDB subclasses. Twelve metabolites had an average relative abundance greater than 100,000, and 64 metabolites had an average relative abundance between 10,000 and 100,000. Among these, 139 metabolites were unclassified, 86 were ‘Lipids and lipid-like molecules’, 42 were ‘Organic acids and derivatives’, 40 were ‘Organic oxygen compounds’, 39 were ‘Organoheterocyclic compounds’, 25 were ‘Nucleosides, nucleotides, and analogues’, and 18 were ‘Phenylpropanoids and polyketides’. Notably, the identified lipids, organic acids, and others are key metabolites constituting the core nutritional components of bovine milk.

#### 3.3.2 Differential milk metabolites DEMs and KEGG enrichment analysis

**Figure 6:**
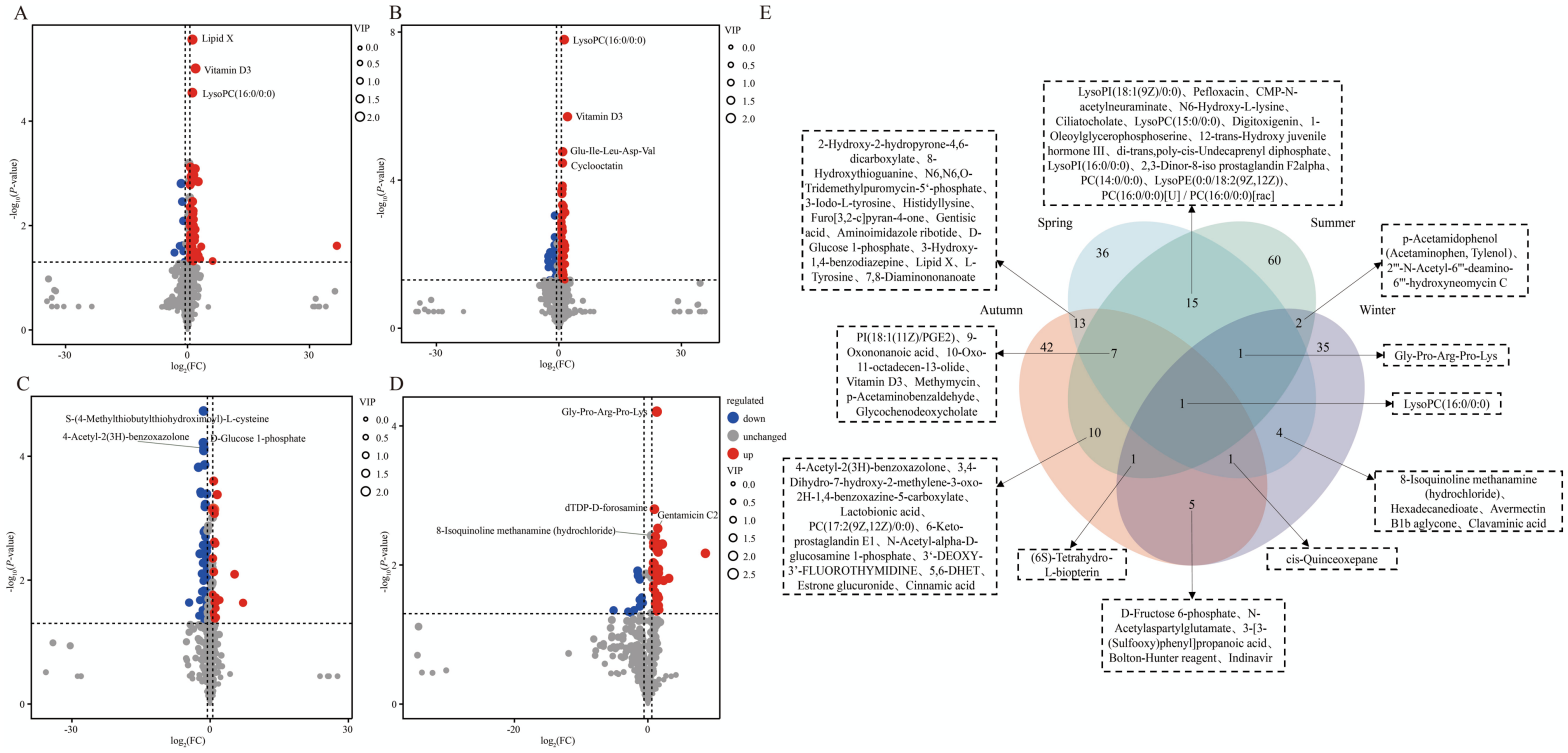
Differential expression characteristics of milk metabolites in XJBC and CHC across different seasons. **(A)** Volcano plot of differential metabolites in milk between two varieties in spring. **(B)** Volcano plot of differential metabolites in milk between two varieties in summer. **(C)** Volcano plot of differential metabolites in milk between two varieties in autumn. **(D)** Volcano plot of differential metabolites in milk between two varieties in winter. **(E)** Venn diagram of differential metabolites common across the four seasons.

To analyze the seasonal differences in the milk metabolome between the two cattle breeds, the overall distribution of samples within the same season was first examined via PCA (Supplementary Figures S2A–D). The results showed a trend of partial separation with overlap between the two breeds across spring, summer, autumn, and winter. To accurately identify breed-specific DEMs, OPLS-DA models were subsequently constructed (Supplementary Figures S2E–H). Model validation confirmed the robustness of the models for each season: both R^2^Y and Q^2^Y values were significantly above 0.5, and permutation tests verified no overfitting (evidenced by a significant drop in permuted R^2^Y and Q^2^Y values and a Q^2^Y regression line intercept < 0). These findings confirm that the models are robust and reliable for subsequent analysis. Based on these models, DEM analysis identified 78 (67 upregulated, 11 downregulated; Supplementary Table S8), 97 (71 upregulated, 26 downregulated; Supplementary Table S9), 80 (24 upregulated, 56 downregulated; Supplementary Table S10), and 50 (39 upregulated, 11 downregulated; Supplementary Table S11) DEMs in spring, summer, autumn, and winter, respectively (Figures 6A–D). Venn diagram analysis revealed that (LysoPC)16:0/0:0 (lysophosphatidylcholine) was the only metabolite that was consistently and significantly different across all four seasons (Figure 6E).

**Figure 7:**
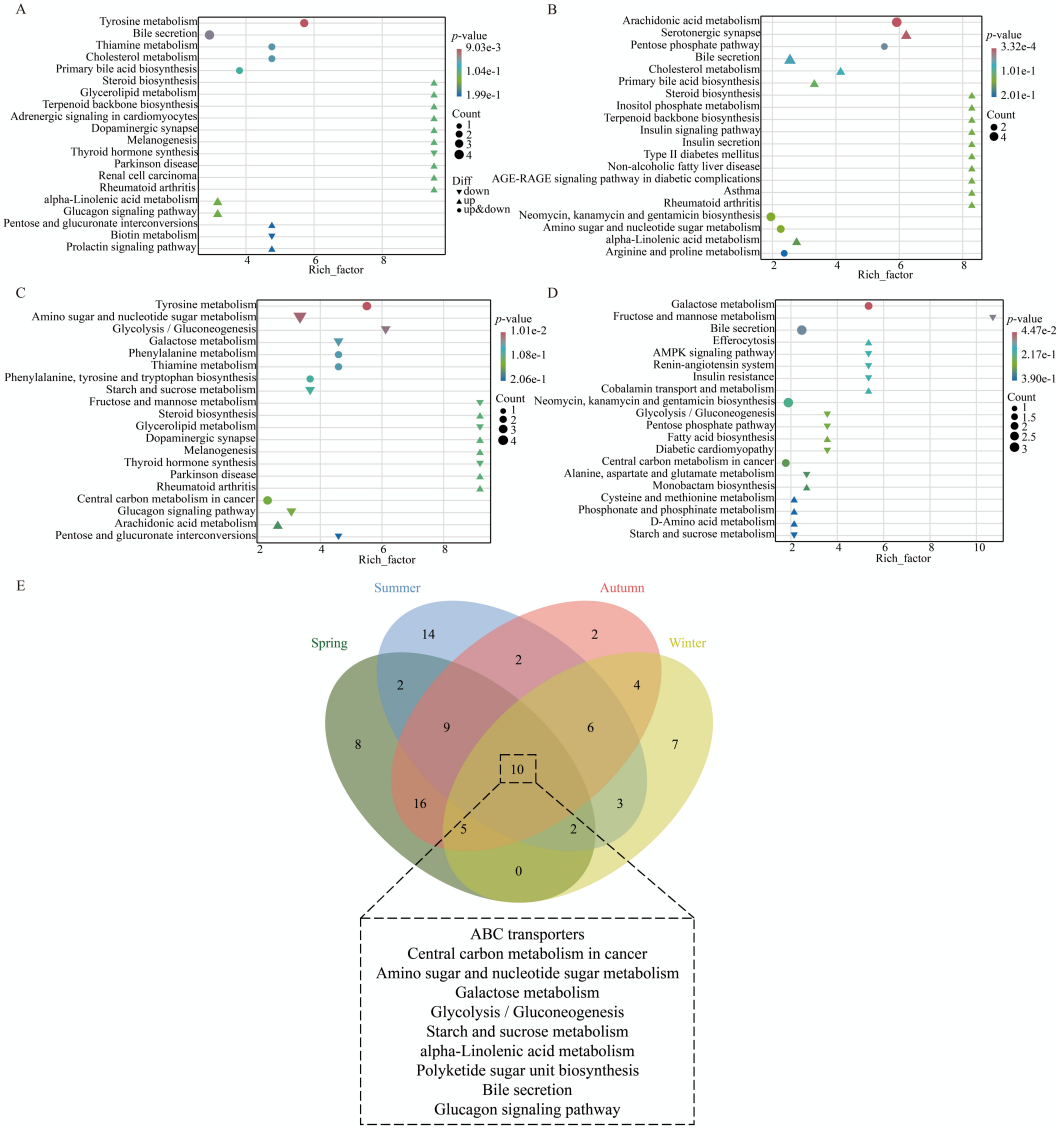
KEGG enrichment analysis of milk metabolites in XJBC and CHC across different seasons. **(A)** KEGG enrichment analysis of DEMs in spring. **(B)** KEGG enrichment analysis of DEMs in summer. **(C)** KEGG enrichment analysis of DEMs in autumn. **(D)** KEGG enrichment analysis of DEMs in winter. **(E)** Common KEGG pathways across different seasons.

Venn diagram analysis (Figure 7E) revealed that the four seasonal comparison groups between Xinjiang Brown cattle and Chinese Holstein cattle shared 10 KEGG pathways, primarily encompassing Galactose metabolism, Glycolysis/Gluconeogenesis, Starch and sucrose metabolism, and alpha-Linolenic acid metabolism, among others. KEGG enrichment analysis indicated that the DEMs in spring were mainly enriched in pathways such as Tyrosine metabolism, Bile secretion, Thiamine metabolism, Cholesterol metabolism, and Primary bile acid biosynthesis. In summer, enrichment was concentrated in pathways including Arachidonic acid metabolism, Serotonergic synapse, Pentose phosphate pathway, Bile secretion, and Cholesterol metabolism. Autumn DEMs primarily involved Tyrosine metabolism, Amino sugar and nucleotide sugar metabolism, Glycolysis/Gluconeogenesis, Galactose metabolism, and Phenylalanine metabolism. Winter DEMs were enriched in pathways such as Galactose metabolism, Fructose and mannose metabolism, Bile secretion, Efferocytosis, and the AMPK signaling pathway (Figures 7A–D; Supplementary Figures S3A–D).

### 3.4 Characteristics of plasma metabolites and KEGG enrichment analysis of DEMs in XJBC and CHC

#### 3.4.1 Overview of plasma metabolites

**Figure 8:**
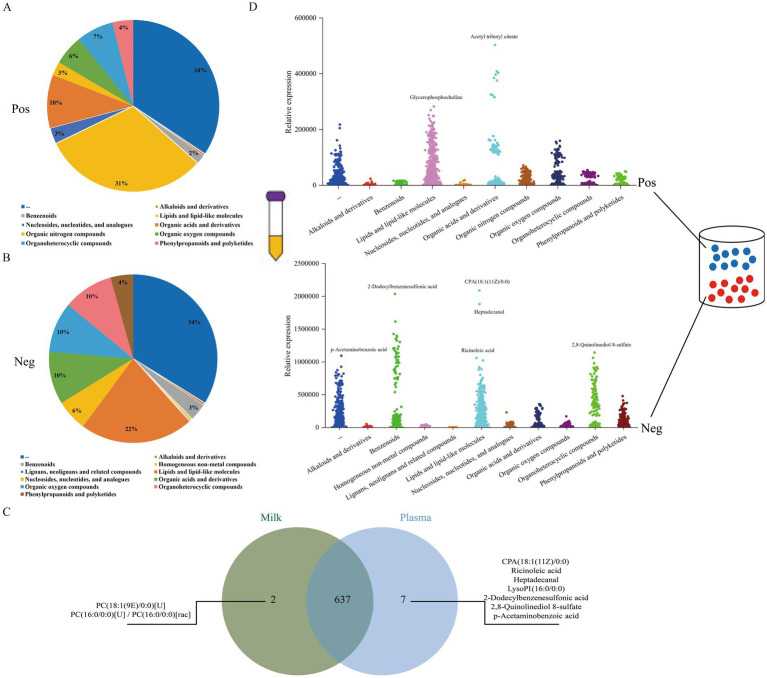
Composition of plasma metabolites in XJBC and CHC. **(A)** Composition of metabolites in positive ion mode. **(B)** Composition of metabolites in negative ion mode. **(C)** Intersection of plasma metabolites and milk metabolites. **(D)** Expression characteristics of plasma metabolites.

Based on the plasma metabolomic data, 644 metabolites were identified. Their category distribution and expression characteristics are shown in Figures 8A,B,D. Among these, 230 metabolites were detected in positive ion mode. These were classified into 10 superclasses, 35 classes, and 56 subclasses according to the HMDB classification. Three metabolites had an average relative abundance greater than 100,000, and 35 metabolites had an average relative abundance between 10,000 and 100,000. Of these, 79 metabolites were unclassified, 72 were ‘Lipids and lipid-like molecules’, and 23 were ‘Organic acids and derivatives’. Additionally, 414 metabolites were detected in negative ion mode. These were classified into 11 HMDB superclasses, 61 HMDB classes, and 93 HMDB subclasses. Nineteen metabolites had an average relative abundance greater than 100,000, and 64 metabolites had an average relative abundance between 10,000 and 100,000. Within this group, 140 metabolites were unclassified, 90 were ‘Lipids and lipid-like molecules’, 42 were ‘Organic acids and derivatives’, 40 were ‘Organic oxygen compounds’, 40 were ‘Organoheterocyclic compounds’, 25 were ‘Nucleosides, nucleotides, and analogues’, and 18 were ‘Phenylpropanoids and polyketides’. Venn diagram analysis was further conducted to compare the milk and plasma metabolomes (Figure 8C). The results revealed that they shared 637 metabolites, indicating a high degree of similarity in their metabolic composition.

#### 3.4.2 Differential plasma metabolites DEMs and KEGG enrichment analysis

**Figure 9:**
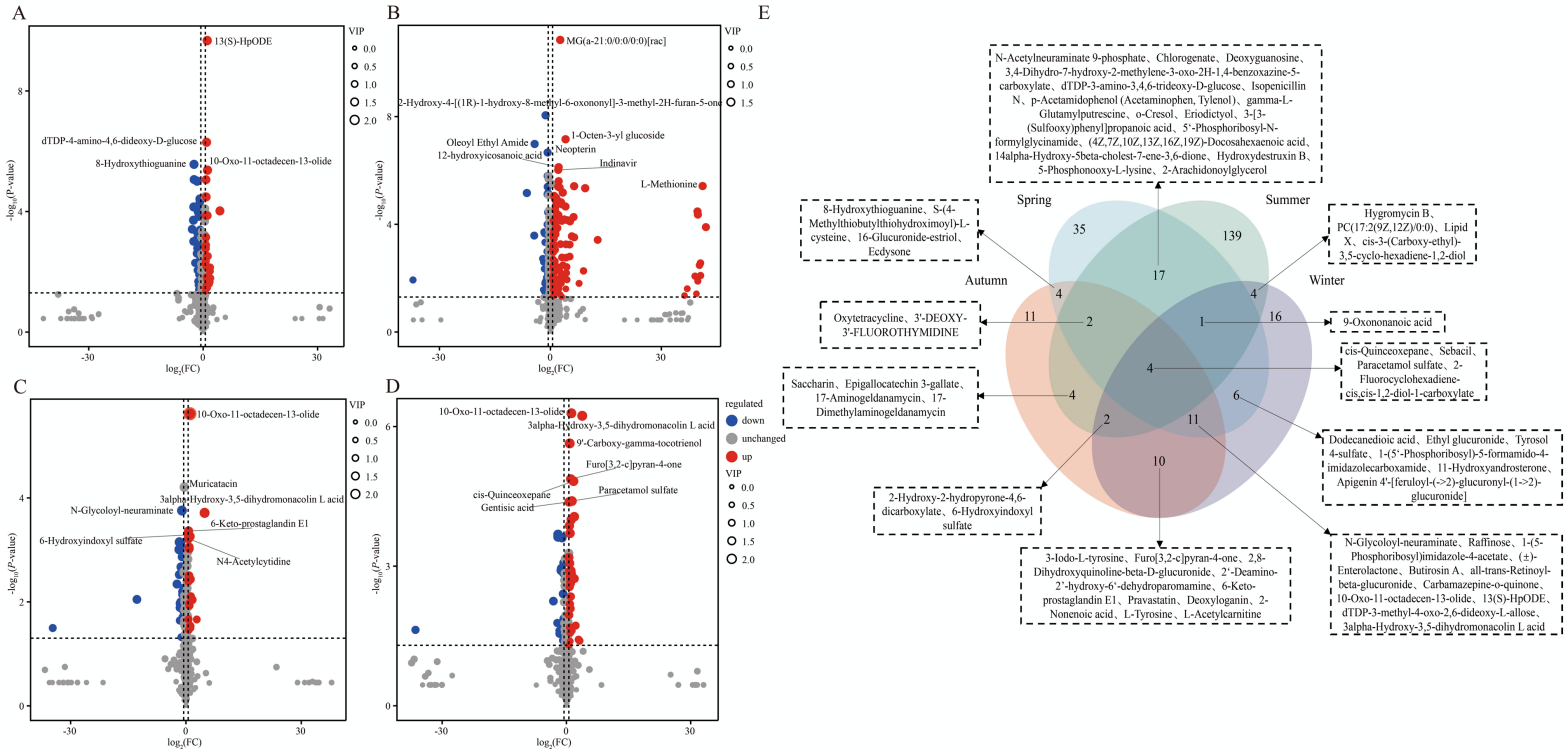
Differential expression characteristics of plasma metabolites in XJBC and CHC across different seasons. **(A)** Volcano plot of plasma differential metabolites between two varieties in spring. **(B)** Volcano plot of plasma differential metabolites between two varieties in summer. **(C)** Volcano plot of plasma differential metabolites between two varieties in autumn. **(D)** Volcano plot of plasma differential metabolites between two varieties in winter. **(E)** Venn diagram of differential metabolites common across the four seasons.

To investigate the seasonal variations in the plasma metabolome between the two breeds, the overall distribution of samples within each season was first assessed using PCA (Supplementary Figures S4A–D). The results indicated partial separation, with some overlap between the two breeds across spring, summer, autumn, and winter. Subsequent OPLS-DA combined with permutation tests confirmed that the models for each season were robust and reliable, showing no signs of overfitting, thus validating their suitability for screening differential metabolites (Supplementary Figures S4E–H). Based on these validated models, DEM analysis identified 80 (32 upregulated, 48 downregulated; Supplementary Table S12), 173 (115 upregulated, 58 downregulated; Supplementary Table S13), 48 (24 upregulated, 24 downregulated; Supplementary Table S14), and 54 (34 upregulated, 20 downregulated; Supplementary Table S15) DEMs in spring, summer, autumn, and winter, respectively (Figures 9A–D). Venn diagram analysis revealed that cis-Quinceoxepane, Sebacil, Paracetamol sulfate, and 2-Fluorocyclohexadiene-cis,cis-1,2-diol-1-carboxylate were the differential metabolites common to all four seasons (Figure 9E).

**Figure 10:**
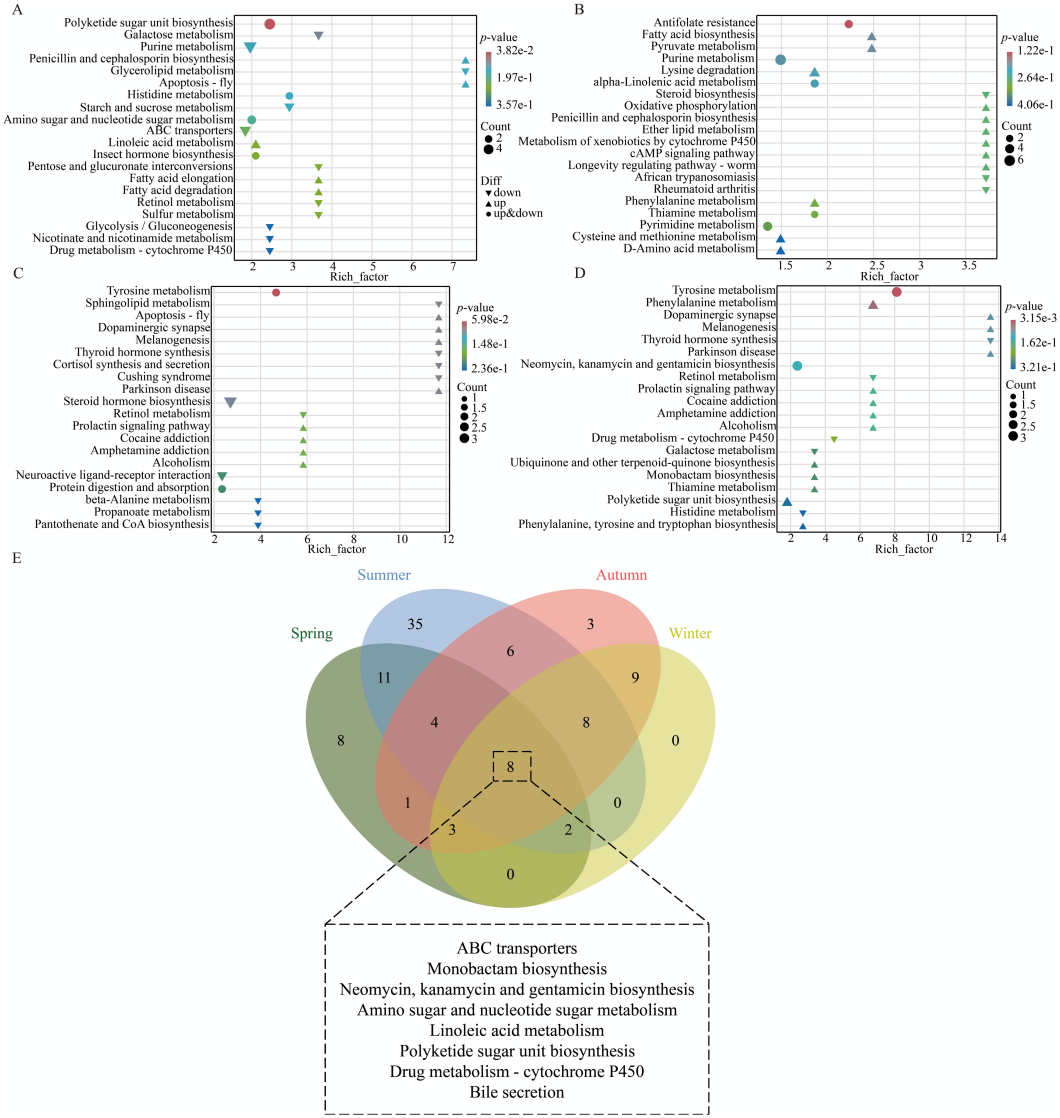
KEGG enrichment analysis of plasma metabolites in XJBC and CHC across different seasons. **(A)** KEGG enrichment analysis of DEMs in spring. **(B)** KEGG enrichment analysis of DEMs in summer. **(C)** KEGG enrichment analysis of DEMs in autumn. **(D)** KEGG enrichment analysis of DEMs in winter. **(E)** Common KEGG pathways across different seasons.

It is noteworthy that 8 common KEGG pathways were identified from plasma metabolite differences between the two breeds. These primarily included ABC transporters, Monobactam biosynthesis, Neomycin, kanamycin, and gentamicin biosynthesis, Amino sugar and nucleotide sugar metabolism, and Linoleic acid metabolism, among others (Figure 10E). KEGG analysis indicated (Figures 10A–D; Supplementary Figures S5A–D) that the spring DEMs were mainly enriched in pathways such as Polyketide sugar unit biosynthesis, Galactose metabolism, Purine metabolism, Penicillin and cephalosporin biosynthesis, and Glycerolipid metabolism. The summer DEMs were concentrated in pathways including Antifolate resistance, Fatty acid biosynthesis, Pyruvate metabolism, Purine metabolism, and Lysine degradation. Autumn DEMs were primarily enriched in Tyrosine metabolism, Sphingolipid metabolism, Apoptosis-fly, Dopaminergic synapse, and Melanogenesis. Winter DEMs mainly involved pathways such as Tyrosine metabolism, Phenylalanine metabolism, Dopaminergic synapse, Melanogenesis, and Thyroid hormone synthesis.

### 3.5 Integrated analysis of DEGs and DEMs

Utilizing bidirectional O2PLS for the integrative analysis of blood transcriptomic data with milk/plasma metabolomic data revealed significant associations between genes and metabolites (Supplementary Figures S6A–H). Pearson correlation analysis was performed between the top 20 DEGs and the top 20 DEMs in milk/plasma for each season. The results indicated that the majority of DEGs and DEMs exhibited significant correlations. At the blood-milk metabolic axis (Supplementary Figures S7A–D), in spring, genes such as *ADGRV1* and *FAT1* showed positive correlations with metabolites like Clavaminic acid, and negative correlations with others such as 3-Hydroxy-1,4-benzodiazepine. In summer, genes such as *IL12B* correlated positively with metabolites, including LysoPC(16:0/0:0). In autumn, *SSPO* correlated positively with specific nucleotide metabolites. In winter, *DPYSL3* correlated positively with cis-Quinceoxepane. A similar season-specific pattern of association was observed at the blood-plasma metabolic axis (Supplementary Figures S7E–H). For instance, in spring, *CCDC194* showed a positive correlation with Carbamazepine-o-quinone, whereas in winter, genes such as *SSPO* showed strong positive correlations with the same metabolite.

**Figure 11:**
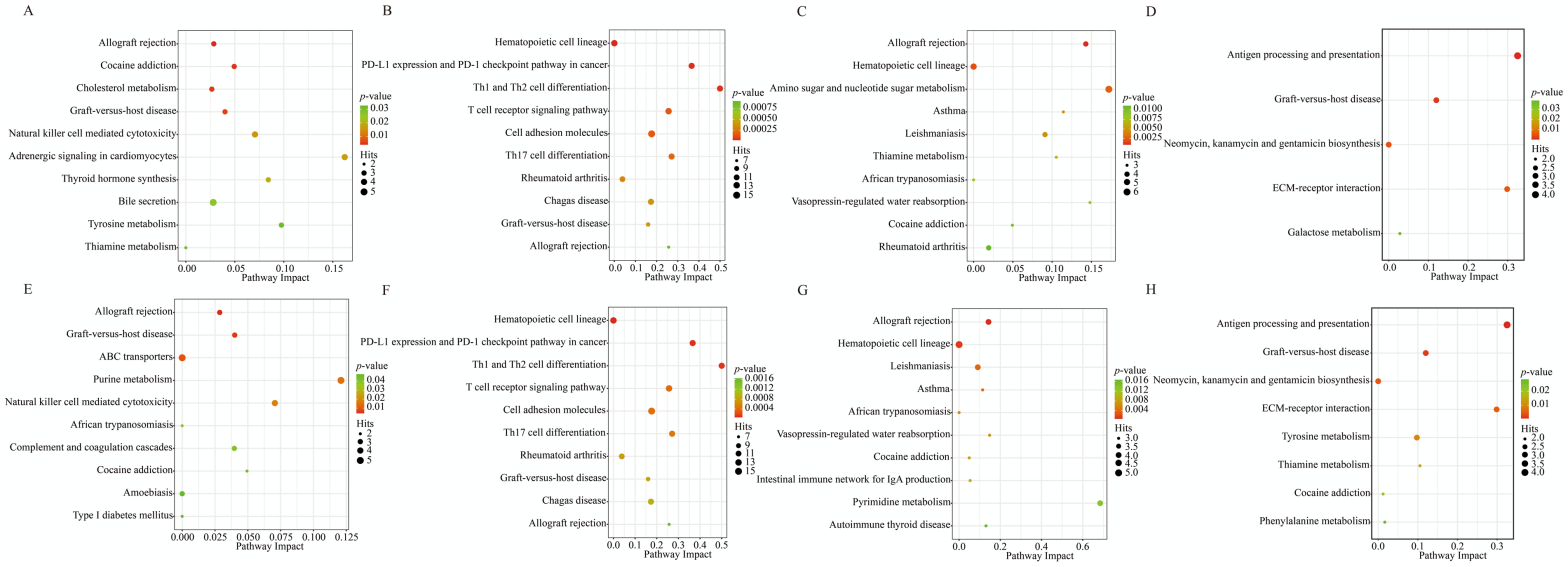
KEGG co-enrichment analysis of DEGs and DEMs in XJBC and CHC across different seasons. **(A)** Joint enrichment analysis of blood transcriptome and milk metabolome in spring. **(B)** Joint enrichment analysis of blood transcriptome and milk metabolome in summer. **(C)** Joint enrichment analysis of blood transcriptome and milk metabolome in autumn. **(D)** Joint enrichment analysis of blood transcriptome and milk metabolome in winter. **(E)** Joint enrichment analysis of blood transcriptome and plasma metabolome in spring. **(F)** Joint enrichment analysis of blood transcriptome and plasma metabolome in summer. **(G)** Joint enrichment analysis of blood transcriptome and plasma metabolome in autumn. **(H)** Joint enrichment analysis of blood transcriptome and plasma metabolome in winter.

It should be noted that the KEGG co-enrichment analysis was performed based on the differential molecules between the two breeds within each season; therefore, the co-enriched pathways presented here are common to both breeds as they reflect the functional characteristics of breed differences across seasons. KEGG co-enrichment analysis revealed dynamic adaptive patterns. At the blood-milk metabolic axis (Figures 11A–D), a significant co-enrichment of 16, 44, 21, and 5 pathways was observed in spring, summer, autumn, and winter, respectively. In spring, DEGs and DEMs were significantly co-enriched in pathways such as Thyroid hormone synthesis, Tyrosine metabolism, Cholesterol metabolism, and Purine metabolism, primarily involved in mammary gland development, fundamental metabolism, and nutrient transport. In summer, they were significantly co-enriched in pathways including the NF-kappa B signaling pathway, the Arachidonic acid metabolism, the T cell receptor signaling pathway, and the Chemokine signaling pathway. These pathways collectively regulate mammary immune homeostasis under heat stress and maintain lactation health by coordinating the inflammatory microenvironment and immune clearance functions. In autumn, significant co-enrichment was found in pathways such as Amino sugar and nucleotide sugar metabolism, Purine/Pyrimidine metabolism, and Phenylalanine, tyrosine, and tryptophan biosynthesis, which are related to nutrient conversion and milk synthesis, and participate in lactose precursor supply, milk water regulation, and nucleic acid synthesis. In winter, DEGs and DEMs associated with energy utilization and mammary structure maintenance were significantly co-enriched in pathways such as Galactose metabolism, ECM-receptor interaction, and Antigen processing and presentation.

At the blood-plasma metabolic axis (Figures 11E–H), a significant co-enrichment of 10, 37, 23, and 8 pathways was observed in the four seasons, respectively. These pathways are related to systemic nutrient supply, stress adaptation, and support for mammary function. In spring, DEGs and DEMs were significantly co-enriched in pathways including ABC transporters, Purine metabolism, and Complement and coagulation cascades, involved in nutrient transport, nucleotide precursor supply for mammary cell proliferation, and immune defense. In summer, they were significantly co-enriched in pathways such as the NF-kappa B signaling pathway, the T cell receptor signaling pathway, and the cGMP-PKG signaling pathway, which are involved in maintaining vascular endothelial integrity, regulating inflammatory responses, and stabilizing the lactation system under heat stress. In autumn, DEGs and DEMs were significantly co-enriched in pathways like Thyroid hormone synthesis, Cortisol synthesis and secretion, Pyrimidine metabolism, and Sphingolipid metabolism. These are associated with neuroendocrine stress adaptation, regulation of systemic water-electrolyte balance, and sphingolipid metabolism (a key component of the milk fat globule membrane), collectively participating in metabolic rebalancing and the preparation of milk component precursors during the seasonal transition. In winter, they were significantly co-enriched in pathways including Tyrosine metabolism, Phenylalanine metabolism, and ECM-receptor interaction, which are related to the directed supply of aromatic amino acids required for milk protein synthesis in cold seasons and the maintenance of the structural integrity of basement membranes in tissues like the mammary gland.

## 4 Discussion

By integrating blood transcriptome data with milk/plasma metabolome data, this study elucidated the molecular adaptation characteristics of Xinjiang Brown cattle and Chinese Holstein cattle across four seasons. Through multi-omics integrative analysis, key regulatory networks associated with lactation function were constructed, providing molecular-level clues for understanding the link between physiological regulation and lactation performance in the two breeds. In this study, only seven individuals were included per breed, and the relatively limited sample size may affect statistical power. However, all individuals were from the same cohort of experimental cattle, and the longitudinal design with repeated sampling across four seasons can, to some extent, control for individual variation, thereby enhancing the reliability of the results. In recent years, transcriptome sequencing technology has been widely applied in livestock and poultry research, playing a crucial role in screening DEGs and exploring gene functions, offering a powerful tool for deciphering the molecular mechanisms underlying traits such as growth, reproduction, and disease resistance (19). This study analyzed DEGs across the four seasons in Xinjiang Brown cattle and Chinese Holstein cattle. The results showed 124 DEGs (76 upregulated, 48 downregulated) in spring, 877 DEGs (565 upregulated, 312 downregulated) in summer, 169 DEGs (86 upregulated, 83 downregulated) in autumn, and 81 DEGs (36 upregulated, 45 downregulated) in winter. Notably, the number of differential genes in summer far exceeded that in other seasons. These DEGs included multiple genes known to be associated with immunity, metabolism, and mammary gland function, such as *IGFBP7*, *FAT1*, *SELP*, and *CCL5*. Based on their differential expression patterns and existing literature, this study proposes the following hypotheses regarding their potential functions in lactation. For instance, research by Evdokimova et al. (20) found that Insulin-like growth factor-binding protein 7 (*IGFBP7*) is a key secreted protein that regulates cell growth and metabolism, and its expression may modulate the proliferation and metabolism of mammary epithelial cells. *FAT1*, a member of the cadherin family, is crucial for maintaining the barrier function of mammary epithelial tissue by regulating epithelial cell polarity and intercellular junctions (21). *CCL5*, a key inflammatory factor, was downregulated in heat-tolerant dairy cows, suggesting that heat-tolerant individuals may mitigate heat-stress-induced damage by suppressing chemokine-mediated inflammatory responses (22). This gene serves as an inflammatory marker in dairy cows for mastitis and endometritis, and also shows significant expression changes during heat stress and lipid metabolism processes (22, 23). Studies have shown that in the healthy lactating mammary gland, *CCL5* is involved in the construction of the immunoregulatory network, and its expression level is closely associated with the maintenance of local immune homeostasis (24). The *PLA2G4A* gene encodes a key enzyme for arachidonic acid synthesis, playing an important role in growth and adipocyte differentiation (25). Arachidonic acid is a significant *ω*-6 polyunsaturated fatty acid that plays a key role in the occurrence and development of mastitis in dairy cows. Its metabolites, particularly prostaglandins and leukotrienes, can regulate immune and inflammatory responses in the mammary gland (26). *IGFBP7* showed differential expression again in the autumn group, suggesting it may still play an important regulatory role in the mid-to-late lactation stage, potentially affecting metabolic adaptation and renewal of mammary cells. The consistent differential expression of *FAT1* further supports its functional importance in maintaining mammary epithelial integrity. Furthermore, Marginal Zone B and B1 Cell Specific Protein (*MZB1*) is a B-cell-specific encoded protein that can protect glucose-regulated proteins and B-lymphocyte-induced maturation proteins, regulate calcium balance, promote antibody production, and facilitate integrin activation in B cells (27, 28). *TREM1* was identified as a key gene significantly upregulated in *E. coli*-induced bovine mastitis, and its high expression is closely associated with severe inflammatory damage in mammary tissue and with significantly elevated apoptosis (29).

Xinjiang Brown cattle and Chinese Holstein cattle are important dairy sources in the Xinjiang region. Further enhancing the economic value or milk production performance of their milk is a key objective in the breeding programs for these two breeds. However, the current understanding of milk metabolite composition in these two breeds remains quite limited, which, to some extent, constrains the in-depth development of their molecular breeding. Existing research has confirmed that metabolomics using LC–MS/MS (Liquid chromatography–tandem mass spectrometry) is a reliable method for analyzing milk composition (30, 31). Therefore, this study first used LC–MS/MS to systematically construct milk metabolite expression profiles of Xinjiang Brown cattle and Chinese Holstein cattle across different seasons, followed by corresponding statistical analysis. In addition to basic components such as water, lactose, fat, protein, carbohydrates, and vitamins, milk also contains a rich array of metabolites. The expression profiles constructed in this study identified numerous highly expressed metabolites, further enriching our understanding of milk composition and illustrating that the expression of milk metabolites is also a crucial component reflecting bovine lactation performance. A total of 232 and 407 milk metabolites were identified in positive and negative ion modes, respectively. The main categories included lipids and lipid-like molecules, organic acids and derivatives, organoheterocyclic compounds, organic oxygen compounds, phenylpropanoids and polyketides, among others. Specifically, 72 and 86 lipids and lipid-like molecules were identified in positive- and negative-ion modes, respectively. In contrast, 23 and 42 organic acids and their derivatives were identified, highlighting the importance of lipid and organic acid metabolism in milk composition. Lipids in milk are core nutritional components, consisting of phospholipids, cholesterol, and triglycerides, providing primary energy, essential fatty acids, and bioactive lipid signaling molecules for neonates (32). The categories of metabolites identified here are similar to the basic metabolite compositions reported in camel milk, Holstein cow milk, buffalo milk, yak milk, donkey milk, horse milk, goat milk, and cashmere goat milk (33). It was found that the milk metabolites with high relative abundance detected in positive ion mode were primarily various PCs (e.g., PC (16:0/0:0)[U]/PC(16:0/0:0)). Compared to positive mode, negative mode contained PS (e.g., PS (18:1(9Z)/0:0)) with a higher average relative abundance. Previous studies indicate that the main phospholipids in human milk are PE (Phosphatidylethanolamine), PC (Phosphatidylcholine), and sphingomyelin, with small amounts of PS (Phosphatidylserine) and PI (Phosphatidylinositol) also present (34). Seasonal differential metabolite profiles were constructed for the milk of Xinjiang Brown cattle and Chinese Holstein cattle. DEM analysis revealed that LysoPC (16:0/0:0) was a common metabolite present across all four seasons. LysoPC is a lysophosphatidylcholine. Previous research shows that LysoPC is a pro-inflammatory lipid with various biological activities produced during pathological processes (35). By acting on endothelial cells, smooth muscle cells, and various immune cells, LysoPC impairs vasodilation function and activates pro-inflammatory signaling pathways (36). In addition to acting as a pro-inflammatory lipid, LysoPC is also extensively involved in membrane repair and serves as a key intermediate in phospholipid metabolism. It can function as a second messenger, activating the MAPK signaling pathway and participating in the regulation of intracellular calcium signaling. Furthermore, it modulates protein kinase activity, thereby influencing cell proliferation, differentiation, and migration (37–39). Notably, this metabolite exhibited significant breed-specific differences between the two breeds. Its expression level was consistently higher in Chinese Holstein cattle across all four seasons, suggesting inherent differences in lysophospholipid metabolism and inflammatory regulation capacity. The persistently higher expression of LysoPC in Chinese Holstein cattle may be associated with their need for inflammatory regulation under high metabolic load, suggesting that attention should be paid to immune balance in breeding; whereas the lower LysoPC level in Xinjiang Brown cattle reflects their stable immune status. The differential accumulation of various membrane lipids and signaling molecules such as LysoPCs, LysoPIs, and DGs (Diglycerides) was observed, including: LysoPC (15:0/0:0), LysoPC (18:2(9Z,12Z)/0:0), LysoPC (16:0/0:0), LysoPI (16:0/0:0), LysoPI (18:1(9Z)/0:0), DG (8:0/20:3(8Z,11Z,14Z)-2OH (5,6)/0:0), etc. Notably, in both spring and summer, the expression levels of LysoPI (16:0/0:0) and LysoPI (18:1(9Z)/0:0) were also significantly higher in Chinese Holstein cattle than in Xinjiang Brown cattle, potentially suggesting a stronger anti-inflammatory regulatory capacity in this breed to maintain mammary gland immune homeostasis under heat stress conditions. Regardless of spring or autumn, the detected DG metabolites (e.g., DG (8:0/20:3(8Z,11Z,14Z)-2OH (5,6)/0:0) and DG (14:0/18:1(11Z)/0:0)) also showed significantly higher expression levels in Chinese Holstein cattle than in Xinjiang Brown cattle, indicating that Chinese Holstein cattle may possess breed-specific characteristics in diacylglycerol metabolism and inflammatory regulation. Numerous studies suggest that PC and DG diglyceride may play unique roles in anti-inflammatory processes (40, 41), while other research indicates that PI molecules play significant roles in inflammation and disease development (42). Among the aforementioned differential metabolites, vitamin D3 (cholecalciferol) is particularly noteworthy and can serve as a key marker reflecting the seasonal adaptation and lactation performance differences between the two cattle breeds. VD3 exhibits significant breed-based differential expression in spring, summer, and autumn. This dynamic pattern suggests its involvement in the spatiotemporal regulation of multiple physiological processes. VD3 is not only a hormone precursor that regulates calcium–phosphorus metabolism and bone health but also acts locally as an immunomodulator and cellular signaling molecule, participating in mammary gland development, immune homeostasis, and stress adaptation through endocrine or paracrine pathways (43). In livestock, the demand for VD3 is extremely high during late gestation and lactation (44). Of note, VD3 is an indispensable nutrient, playing fundamental roles in calcium homeostasis, immune regulation, and mammary function. Thus, the seasonal breed differences in VD3 suggest that Chinese Holstein cattle should focus on immune regulation under summer heat stress, while Xinjiang Brown cattle need to pay attention to calcium metabolism and immune reserves in spring and autumn. Therefore, VD3 is not only a key factor in nutritional metabolism but also a molecular marker of lactation performance in the two cattle breeds. Prostaglandin A2 is a class of bioactive lipid mediators involved in inflammation and cellular signaling (45). D-Glucose 1-phosphate is the direct precursor for lactose synthesis, while D-Fructose 6-phosphate may provide the carbon skeleton via gluconeogenesis (46). It is hypothesized that these two metabolites may be key nodes in the lactose synthesis pathway. Ten common KEGG pathways were identified across all four seasons, including ABC transporters, Amino sugar and nucleotide sugar metabolism, Galactose metabolism, Glycolysis/Gluconeogenesis, Starch and sucrose metabolism, and the Glucagon signaling pathway, among others. These pathways may be involved in regulating the lactation performance of the two cattle breeds. ABC transporters are proteins that mediate the active transmembrane transport of various molecules. In the mammary gland, they transport substrates such as inorganic salts, amino acids, nucleotides, and polysaccharides from the blood into epithelial cells, providing precursors for the synthesis of milk fat, milk protein, and lactose (47). Differentially expressed metabolites such as Cytidine, D-Glucose, L-Phenylalanine, and Raffinose were enriched in the ABC transporters pathway. Therefore, it is speculated that these DEMs may regulate the synthesis of major milk nutrients across different seasons via this pathway. Starch and sucrose in forage and feed are broken down into monosaccharides in cattle, providing direct precursors for lactose synthesis (8). These monosaccharides are further metabolized through pathways such as glycolysis, generating ATP and reducing power to supply energy and metabolic substrates for the synthesis of milk fat, milk protein, and lactose (48). In this study, D-glucose 1-phosphate, D-glucose, and D-fructose 6-phosphate were all enriched in pathways such as glycolysis/gluconeogenesis, starch and sucrose metabolism, and galactose metabolism, indicating that these carbohydrate metabolites may participate in the regulation of milk component synthesis through the aforementioned pathways. In addition, metabolites such as CMP-N-acetylneuraminate and N-glycoloylneuraminate, which were enriched in the amino sugar and nucleotide sugar metabolism pathway, may be involved in regulating the physiological functions of mammary epithelial cells.

Blood plays a crucial role in milk synthesis, serving functions such as transporting nutrients and hormones, clearing metabolic waste, and providing immune protection (8). Plasma is the key component of blood responsible for these functions. This complex medium, composed of water, proteins, lipids, inorganic salts, and various small-molecule metabolites, serves not only as the medium for the exchange of materials between blood and cells/tissues but also as the metabolic environment for blood cells. Consequently, its composition is constantly in flux. Plasma metabolites are important biomarkers reflecting the physiological state of the body, and changes in their expression have been confirmed to be closely associated with various pathological conditions such as cancer and inflammation (49). However, there is currently a lack of systematic understanding regarding the dynamic relationship between plasma metabolite expression and bovine lactation performance under healthy lactating conditions. Therefore, investigating the impact of plasma metabolite expression on lactation performance, mainly when analyzed in conjunction with seasonal environmental factors, is not only significant for cattle management, mastitis diagnosis, and treatment, but also provides an important theoretical foundation and practical basis for improving lactation performance from the perspective of plasma metabolites. In positive and negative ion modes, 230 and 414 metabolites were identified from plasma, respectively. Their main categories were similar to those identified in milk. Specifically, 72 and 90 lipids and lipid-like molecules were identified in positive- and negative-ion modes, respectively. In contrast, 23 and 42 organic acids and their derivatives were identified, highlighting the importance of lipid and organic acid metabolism in plasma composition. There were 637 shared metabolites between plasma and milk, indicating a similarity in the elemental composition of their metabolomes, although specific metabolite types differed. Among these, lipids and lipid-like molecules showed high abundance in both plasma and milk. Notably, plasma was enriched with more metabolites closely related to core physiological and pathological processes, including lipid metabolism, energy metabolism, inflammation, and oxidative stress. DEM analysis revealed differences in plasma metabolite expression across seasons, with 80, 173, 48, and 54 DEMs identified in the spring, summer, autumn, and winter groups, respectively. This study found that phospholipid molecules detected in the plasma metabolites of the spring, summer, and winter groups included PE and PC. Examples include PE (15:0/P-18:1(11Z)), PC (17:2(9Z,12Z)/0:0), and PC (17:2(9Z,12Z)/0:0), MG (15:0/0:0/0:0), MG (19:0/0:0/0:0), and MG (a-21:0/0:0/0:0) were screened in the spring and summer groups. According to Tan et al. the MG (Monoglyceride) metabolite was found to be negatively correlated with pro-inflammatory cytokines and positively correlated with anti-inflammatory cytokines (50). The summer group contained a small number of LysoPI (Lyso-phosphatidylinositol), LysoPE (Lyso-phosphatidylethanolamine), and LysoPA (Lyso-phosphatidic acid), all of which belong to lysophospholipids, such as LysoPI (16:0/0:0), LysoPI (18:1(9Z)/0:0), LysoPA (0:0/18:2(9Z,12Z)), and LysoPE (0:0/18:2(9Z,12Z)). In contrast to the consistent pattern observed in milk metabolites, breed-specific differences in plasma metabolites were more diverse, varying by metabolite type and season. For example, in spring, MG (15:0/0:0/0:0) and MG (19:0/0:0/0:0) were higher in Chinese Holstein cattle, while PE (15:0/P-18:1(11Z)) was higher in Xinjiang Brown cattle. In summer, PC (17:2(9Z,12Z)/0:0) and LysoPE (0:0/18:2(9Z,12Z)) were higher in Xinjiang Brown cattle, whereas MG(a-21:0/0:0/0:0)[rac] was higher in Chinese Holstein cattle. In winter, PC (17:2(9Z,12Z)/0:0) was higher in Chinese Holstein cattle. These results indicate that breed differences in plasma metabolites are markedly diverse and seasonal, reflecting complex interactions between genetic background, metabolic pathways, and seasonal adaptation. Notably, the higher expression of LysoPE in Xinjiang Brown cattle during summer may be associated with the breed’s anti-inflammatory capacity. Previous studies indicate that LysoPI has anti-inflammatory effects, LysoPA is involved in inflammatory lung diseases, exhibiting a dual role as both pro- and anti-inflammatory mediators, and LysoPA is also considered a key product of glycolysis and glycerophospholipid metabolism, acting as a mitogen and participating in signal transduction by providing arachidonic acid (51–53). Key metabolites closely associated with milk synthesis, such as Vitamin D3, D-Glucose 1-phosphate, and L-Methionine, which are critical precursors or regulators for calcium homeostasis and immune modulation, lactose synthesis, and milk protein synthesis, respectively, were also identified in the plasma metabolome. Eight common KEGG pathways were found across all four seasons, including ABC transporters and Amino sugar and nucleotide sugar metabolism. The ABC transporters were significantly enriched across all four seasons in both milk and plasma metabolomes, yet the metabolites they transported exhibited marked seasonal specificity, cytidine in spring, D-glucose in summer, L-phenylalanine in autumn, and raffinose in winter, suggesting dynamic changes in the nutritional substrate demands of the mammary gland across different physiological stages. At the breed level, these metabolites showed significant differences between Holstein and Xinjiang Brown cattle. Cytidine in spring, D-glucose in summer, and raffinose in winter were more highly expressed in Holstein cattle, whereas L-phenylalanine in autumn was more highly expressed in Xinjiang Brown cattle, reflecting inherent differences in the mammary nutritional requirements between the two breeds. In plasma, raffinose showed higher expression in Holstein than in Xinjiang Brown cattle across spring, autumn, and winter, consistent with the direction of the difference observed for raffinose in milk during winter, further indicating breed-specific characteristics. These results demonstrate that ABC transporters serve as a core regulatory module, with their fundamental functional network remaining stable across seasons, while the specific transport substrates and their differential expression between breeds constitute the seasonal- and breed-specific regulatory mechanisms. Based on the functional analysis of differential metabolites in milk and plasma described above, it was found that immune-inflammatory regulators, including lysophospholipids such as LysoPC and LysoPI, as well as prostaglandins such as Prostaglandin A2, exhibited significant breed-specific differential expression across the four seasons. Among these, LysoPC (16:0/0:0) was consistently highly expressed in Holstein cattle, suggesting its involvement in regulating mammary gland immune homeostasis and inflammatory responses. Mammary gland energy metabolism regulators, such as carbohydrate metabolites including D-glucose-1-phosphate, D-fructose-6-phosphate, and D-glucose, which serve as direct precursors or key nodal molecules for lactose synthesis, displayed differential expression between breeds, potentially reflecting differences in mammary gland energy utilization efficiency. Regulators of membrane structure and signal transduction, including various membrane lipid molecules such as multiple PC, PS, and DG species, are involved not only in maintaining the structure of the milk fat globule membrane but also act as signaling molecules in cell proliferation, differentiation, and stress responses. Additionally, regulators of calcium homeostasis and immunity, such as Vitamin D3, exhibited sustained breed-specific differential expression in spring, summer, and autumn, and could serve as important markers of seasonal adaptability between the two breeds, participating in calcium-phosphorus metabolism and immune regulation. These findings indicate that the aforementioned categories of metabolites collectively constitute a multidimensional molecular network regulating lactation performance.

Complex molecular networks typically regulate biological processes. While single transcriptomic analysis can identify large numbers of DEGs and related pathways, establishing direct causal relationships is challenging because of the multilevel regulation between genes and the final phenotype. Metabolomics can analyze changes in the types and quantities of metabolites, which form the material basis of an organism’s phenotype. Therefore, in this study, integrating blood transcriptome data with milk/plasma metabolome data from two cattle breeds across different seasons for correlation analysis can bridge gene expression and functional phenotypes, thereby more systematically revealing the regulatory mechanisms underlying lactation performance. It should be noted that the transcriptomic data in this study were obtained from blood, while the metabolomic data were derived from milk and plasma. Although this approach facilitates the elucidation of regulatory networks at the systemic level, blood gene expression reflects systemic physiological status, whereas milk metabolites represent local synthetic activity within the mammary gland. Consequently, the associations between these two datasets may be realized through indirect pathways. Therefore, this study focuses on co-enrichment patterns and functional associations at the molecular level, aiming to provide clues for subsequent tissue-specific validation. To highlight the regulatory mechanisms directly associated with lactation performance, this study focused on three categories of key pathways: lactation-related pathways directly involved in the synthesis and secretion of lactose, milk fat, and milk protein (e.g., galactose metabolism, cholesterol metabolism, and amino sugar and nucleotide sugar metabolism); immunomodulatory pathways mediating mammary gland immune homeostasis and inflammatory responses (e.g., the NF-kappa B signaling pathway, arachidonic acid metabolism, and chemokine signaling pathways); and metabolic adaptation pathways reflecting the strategies of energy metabolism adjustment in response to seasonal changes (e.g., glycolysis/gluconeogenesis and purine/pyrimidine metabolism). Within each season, differentially expressed genes and metabolites were screened through breed comparison (XJBC vs. CHC), followed by KEGG co-enrichment analysis. The enriched pathways reflect both seasonal effects and breed effects. The integrated analysis of the milk metabolome and blood transcriptome indicated that DEGs and DEMs were significantly enriched in multiple key pathways. On one hand, enrichment was observed in pathways directly related to mammary gland development and basic metabolism, including Galactose metabolism, Tyrosine metabolism, Cholesterol metabolism, Amino sugar and nucleotide sugar metabolism, and the Glucagon signaling pathway. Cholesterol is an indispensable structural component of the milk fat globule membrane and an important lipid substance in organisms, participating in the biosynthesis of vitamin D, bile acids, and steroid hormones (54, 55). On the other hand, the analysis also revealed significant enrichment in a series of pathways that play central roles in immune and inflammatory responses, such as the NF-kappa B signaling pathway, Arachidonic acid metabolism, Cytokine-cytokine receptor interaction, T cell receptor signaling pathway, Chemokine signaling pathway, Purine/Pyrimidine metabolism, and Phenylalanine, tyrosine, and tryptophan biosynthesis. These pathways are crucial in immune or anti-inflammatory processes. The NF-kappa B signaling pathway serves as the central hub for initiating and regulating inflammatory responses (56). T cells, as important immune cells (57), are closely associated with purine/pyrimidine metabolism, in which purine metabolism acts as a regulatory hub for immunosuppressive macrophages (58), while pyrimidines exert anti-inflammatory effects (59). Arachidonic acid is not only a key component of milk fat (34), but also a critical pathway in the inflammatory response (60). Collectively, these pathways play important roles in inflammatory responses and the regulation of mammary gland function. Furthermore, in the integrated analysis of the plasma metabolome and transcriptome, pathways such as ECM-receptor interaction, ABC transporters, Cell adhesion molecules, Sphingolipid metabolism, and Phenylalanine metabolism were also significantly enriched. Sphingolipids are important components of animal cell membranes, and their metabolites constitute a class of key bioactive molecules involved in regulating cell growth, differentiation, senescence, and programmed death (61). In milk fat globules, polar lipids are primarily composed of glycerophospholipids and sphingolipids, with sphingomyelin being the predominant form of sphingolipid (62). In this study, the significant enrichment of the sphingolipid metabolism pathway suggests that sphingolipid metabolism may influence lactation performance in the two cattle breeds by affecting the composition and function of the milk fat globule membrane. Notably, the integrated analysis of the milk and plasma omics data co-enriched 51 KEGG pathways. These pathways are primarily involved in key biological processes such as immune-inflammatory responses and mammary gland energy metabolism. Although this enrichment analysis was based on features common to both breeds, further analysis of core molecules within the key pathways revealed that genes enriched in the NF-kappa B signaling pathway, such as *CXCL8* and *DDX58*, exhibited significantly higher expression levels in Chinese Holstein cattle, suggesting that this breed may exhibit a stronger inflammatory response under environmental stimuli such as heat stress. In contrast, the *LCK* gene in the same pathway showed significantly higher expression levels in Xinjiang Brown cattle, potentially reflecting a more stable T cell-mediated adaptive immunoregulation in this breed. Additionally, key genes in the purine metabolism pathway also displayed breed specificity: *AK7* and *ENTPD1* were highly expressed in Chinese Holstein cattle, whereas *ADCY3*, *PDE1B*, *NT5M*, and *ENTPD8* were highly expressed in Xinjiang Brown cattle. The purine metabolite deoxyguanosine showed higher expression in Chinese Holstein cattle in spring but was more highly expressed in Xinjiang Brown cattle in summer, indicating differences between the two breeds in their seasonal response patterns of purine metabolism. These breed-specific differences at the molecular level collectively reveal distinctions between the two breeds in immune-inflammatory regulation and energy metabolism pathways. The O2PLS and Pearson correlation analyses revealed significant associations between several key DEGs and DEMs. For example, in summer, *IL12B* was positively correlated with LysoPC (16:0/0:0) in milk, and given that LysoPC was consistently higher in Chinese Holstein cattle, this correlation suggests a link between *IL12B*-mediated pathways and lysophospholipid metabolism. In winter, *DPYSL3* showed a positive correlation with cis-Quinceoxepane in plasma. These gene–metabolite correlations provide clues for understanding how DEGs regulate lactation function through metabolic pathways, and preliminarily support the potential of DEMs as candidate markers reflecting breed characteristics and physiological status. Based on the above analysis, the breed-specific expression patterns of DEGs and DEMs reveal the divergent molecular strategies employed by the two cattle breeds in seasonal adaptation. Chinese Holstein cattle, having long been selectively bred primarily for high milk production, may possess a relatively weaker capacity to cope with environmental stress. In contrast, Xinjiang Brown cattle, as a dual-purpose breed, have developed a more stable stress adaptation mechanism through long-term ecological adaptation. Such differences in genetic background may directly influence the molecular response patterns observed in this study, resulting in breed-specific characteristics in immune responses, metabolic adaptation, and nutrient transport between the two breeds. Therefore, the transcriptomic and metabolomic differences revealed in this study can be largely attributed to the inherent genetic backgrounds and breeding directions of the two breeds. In summary, key pathways such as NF-kappa B signaling, purine/pyrimidine metabolism, galactose metabolism, and arachidonic acid metabolism may collectively constitute a multi-layered, tightly interactive regulatory network, potentially synergistically modulating lactation performance. The seasonally differentially expressed genes and differentially expressed metabolites identified in this study can serve as candidate targets for subsequent screening of molecular markers associated with high production performance and good environmental adaptability. Furthermore, the metabolic pathways co-enriched in milk and plasma (such as galactose metabolism and ABC transporters) suggest that optimizing energy supply and transmembrane transport efficiency through nutritional regulation may further enhance lactation performance. In summary, the multi-omics regulatory network constructed in this study not only deepens the molecular understanding of seasonal adaptation in cattle but also provides a theoretical basis for precision breeding and refined feeding management, while the identified differentially expressed genes and metabolites, especially those involved in energy metabolism and immune regulation, are expected to serve as molecular markers for selecting individuals with high energy utilization efficiency to enhance economic returns.

## 5 Conclusion

This study integrated blood transcriptome and milk/plasma metabolome data to systematically elucidate the molecular regulatory networks underlying lactation performance and the breed-specific adaptive mechanisms of Xinjiang Brown cattle and Chinese Holstein cattle across different seasons. For each season, we compared the two breeds (XJBC vs. CHC). Therefore, the observed seasonal patterns reflect how breed differences vary across seasons rather than intrinsic seasonal responses of each breed. Transcriptome analysis identified multiple differentially expressed genes potentially involved in lactation-related immune and metabolic regulation; metabolome analysis revealed key differential metabolites that may influence lactation performance through various metabolic pathways. Integrated multi-omics analysis further identified NF-kappa B signaling, purine/pyrimidine metabolism, galactose metabolism, and arachidonic acid metabolism as core pathways co-regulating lactation and immune function. Collectively, this study deciphers the molecular basis of seasonal lactation performance in the two cattle breeds from a multi-omics perspective. The DEGs and DEMs identified herein, particularly those showing significant correlations with each other, represent promising candidate targets for molecular breeding.

## Data Availability

The datasets presented in this study can be found in online repositories. The names of the repository/repositories and accession number(s) can be found at: NCBI repository, accession number: PRJNA1444002, https://www.ncbi.nlm.nih.gov/sra/PRJNA1444002. The metabolomics expression data have been deposited in figshare at: https://doi.org/10.6084/m9.figshare.32033760.

## Footnotes

1
https://cloud.oebiotech.com

2
https://davidbioinformatics.nih.gov/

3
https://www.metaboanalyst.ca/

4
https://www.bioinformatics.com.cn/
