# Supplementary material for: Key regulators of lactation performance in Xinjiang Brown cattle and Chinese Holstein cattle unraveled by multi-omics integration
Source: Front Vet Sci. 2026 May 8;13:1808447. doi: 10.3389/fvets.2026.1808447 (PMC13193985; doi:10.3389/fvets.2026.1808447)
Supplement: Supplementary file 1 [file Data_Sheet_1.PDF]

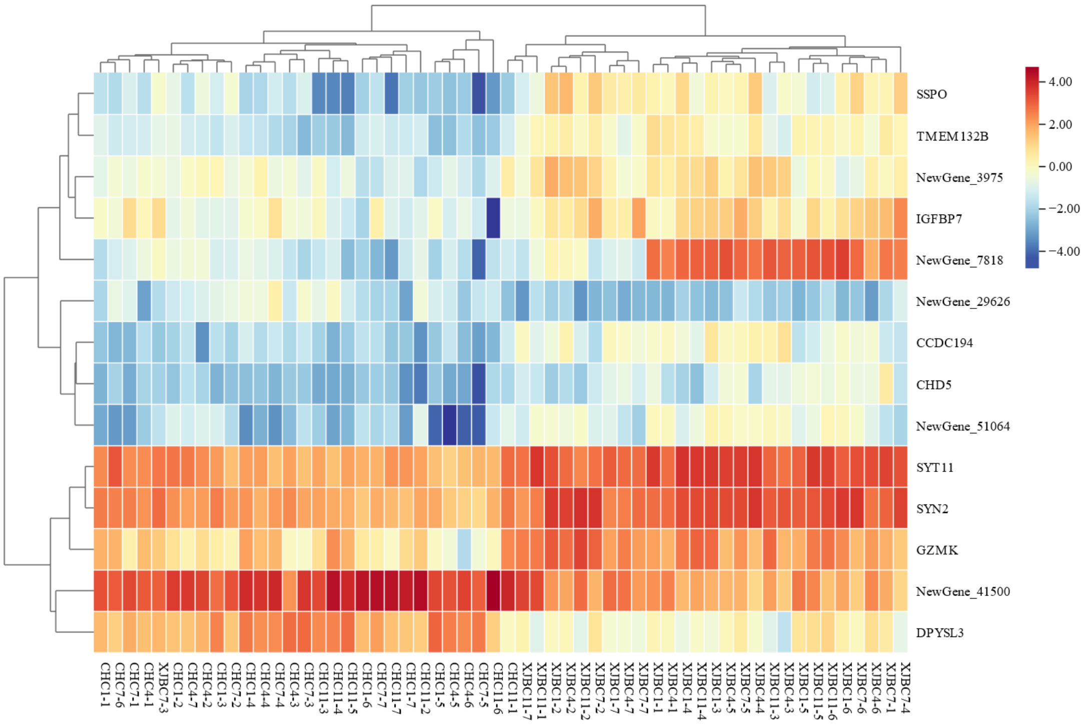

Figure S1. Heatmap of differentially expressed genes common to Xinjiang Brown cattle and Chinese Holstein cattle across four seasons.

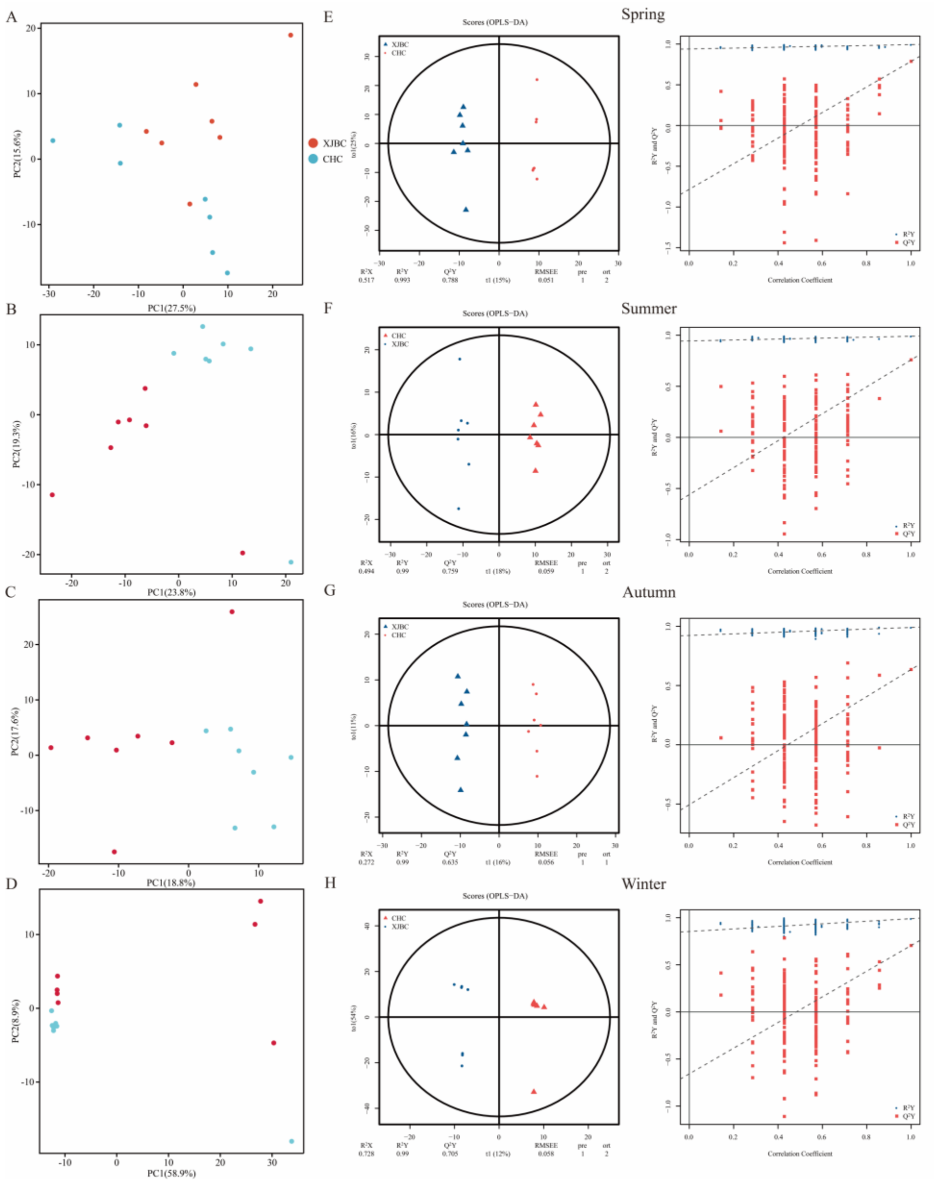

Figure S2. Seasonal differential analysis of milk metabolites in Xinjiang Brown cattle and Chinese Holstein cattle. (A, B, C, D) Principal component analysis score plots of milk metabolite samples across different seasons. (E, F, G, H) Validation plots of Orthogonal Partial Least Squares Discriminant Analysis models for each seasonal group: the left panel shows the OPLS-DA score plot, and the right panel displays the corresponding permutation test plot. Note: In the score plot, the x-axis (t1) represents the predictive component (between-group variation), and the y-axis (to1) represents the orthogonal component (within-group variation). The percentage on the horizontal y-axis indicates the proportion of total variance explained by the corresponding component. The model parameters, including R^2X, R^2Y, Q^2Y, RMSEE (root mean square error of estimation), pre (number of predictive components), and ort (number of orthogonal components), are provided below the figure. In the permutation test plot, the x-axis shows the correlation between permuted groups and the original model groups, and the y-axis represents the values of R^2Y or Q^2Y (where R^2Y and Q^2Y at x = 1 correspond to the original model). Blue and red dots denote the R^2Y and Q^2Y of the permuted models, respectively. The two dashed lines are regression lines fitted for R^2Y and Q^2Y. A positive slope of the Q^2Y regression line indicates that the model is meaningful, and the general distribution of blue dots above red dots suggests good independence between the training and test sets. The same applies below.

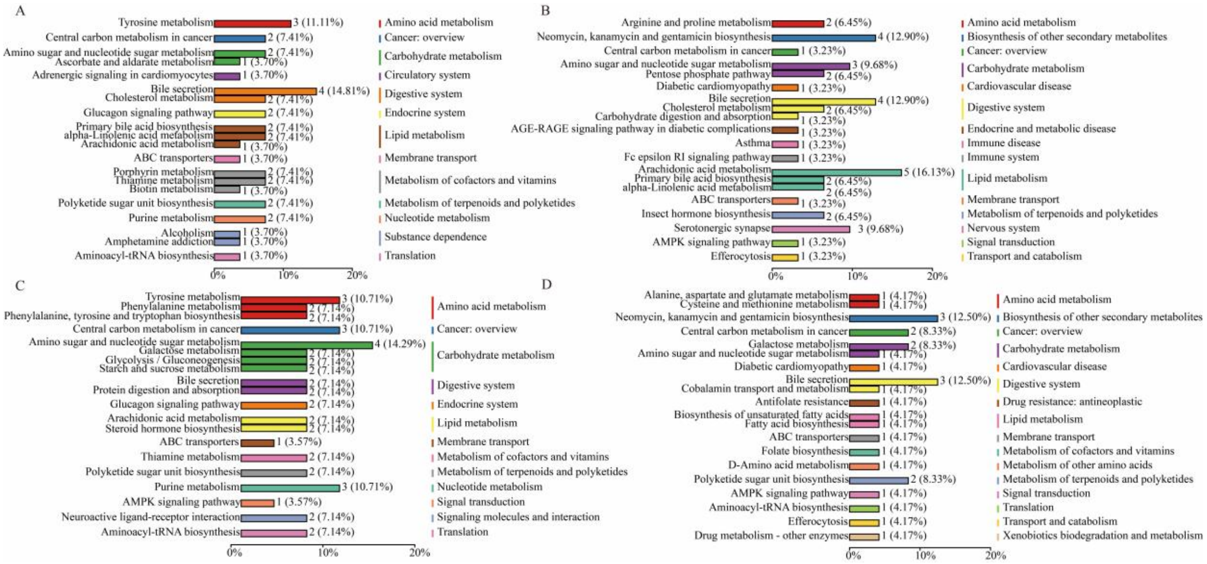

Figure S3. KEGG classification chart of metabolites in milk of Xinjiang Brown cattle and Chinese Holstein cattle in different seasons. (A) Spring. (B) Summer. (C) Autumn. (D) Winter.

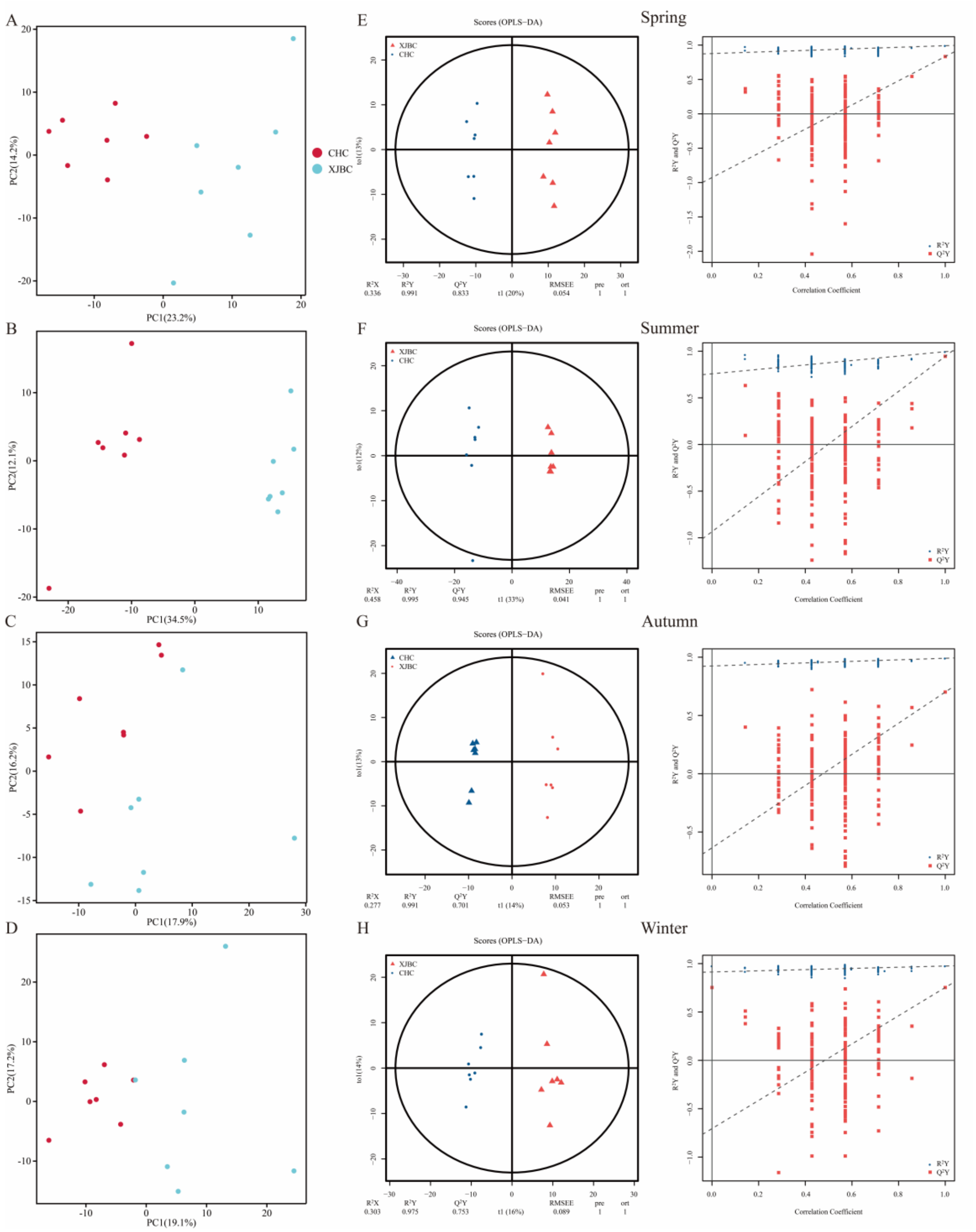

Figure S4. Seasonal differential analysis of plasma metabolites in Xinjiang Brown cattle and Chinese Holstein cattle. (A, B, C, D) Principal component analysis score plots of plasma metabolite samples across different seasons. (E, F, G, H) Validation plots of Orthogonal Partial Least Squares Discriminant Analysis models for each seasonal group: the left panel shows the OPLS-DA score plot, and the right panel displays the corresponding permutation test plot.

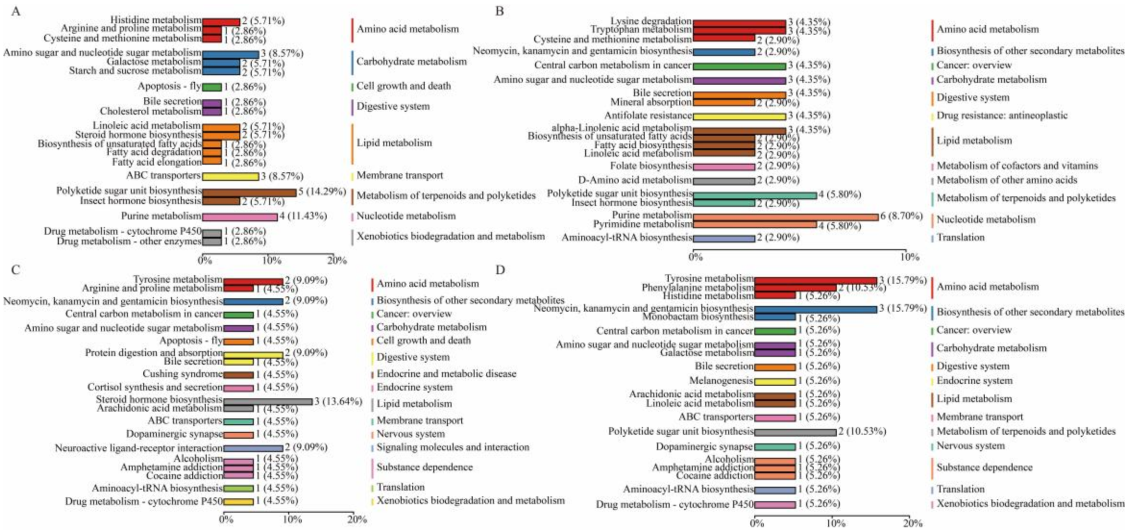

Figure S5. KEGG classification chart of plasma metabolites of Xinjiang Brown cattle and Chinese Holstein cattle in different seasons. (A) Spring. (B) Summer. (C) Autumn. (D) Winter.

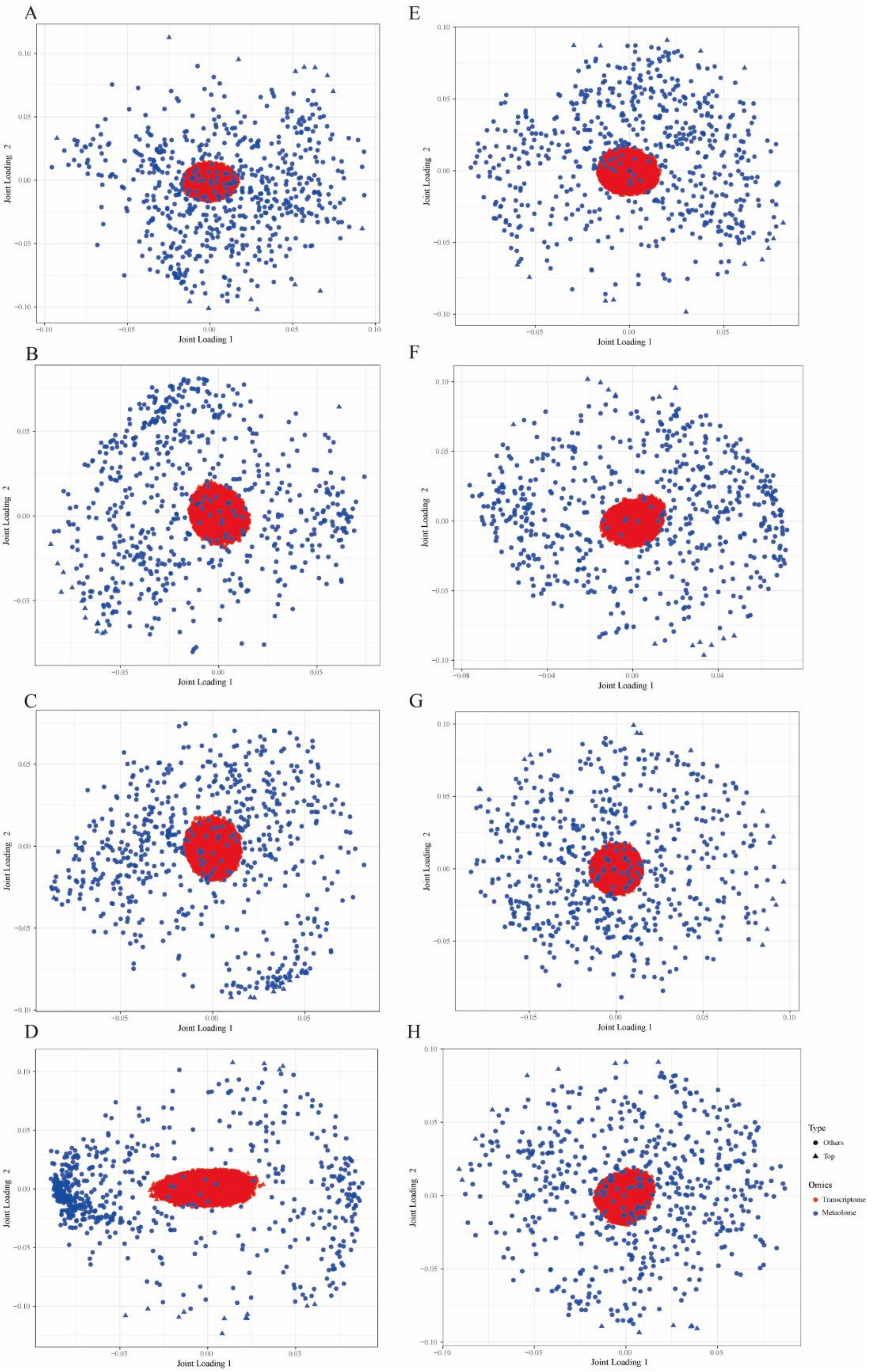

Figure S6. Joint metabolomic and transcriptomic correlation analysis. (A, B, C, D) O2PLS analysis between the blood transcriptome and the milk metabolome across seasons. (E, F, G, H) O2PLS analysis between the blood transcriptome and plasma metabolome across seasons.

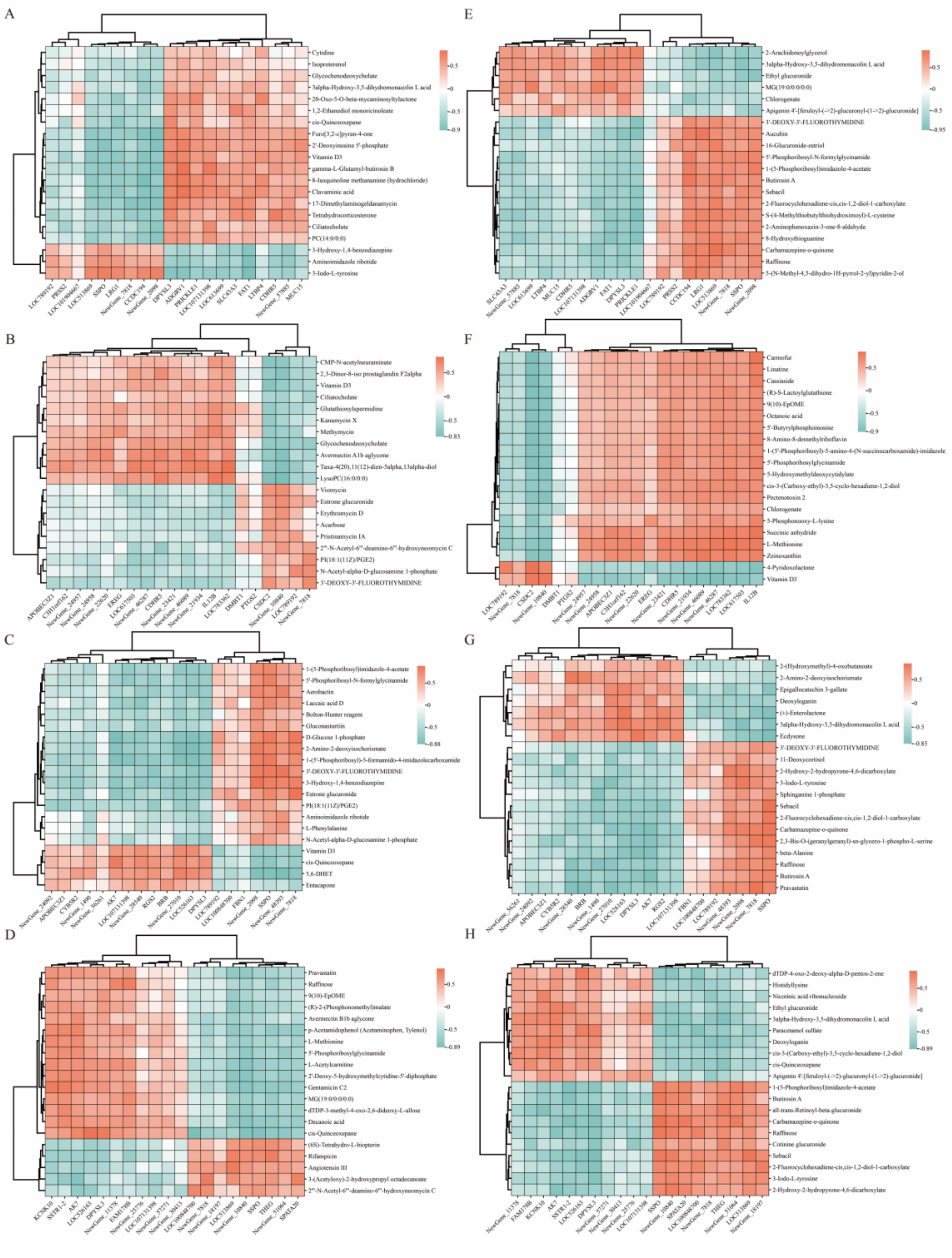

Figure S7. Correlation heatmaps between partial differentially expressed metabolites and differentially expressed genes. (A) Blood transcriptome and milk metabolome. (B) Blood transcriptome and plasma metabolome.
